# Amorphous Poly(ethylene terephthalate) Composites with High-Aspect Ratio Aluminium Nano Platelets

**DOI:** 10.3390/polym14030630

**Published:** 2022-02-07

**Authors:** Arfat Anis, Ahmed Yagoub Elnour, Abdullah Alhamidi, Mohammad Asif Alam, Saeed M. Al-Zahrani, Fayez AlFayez, Zahir Bashir

**Affiliations:** 1 SABIC Polymer Research Center (SPRC), Chemical Engineering Department, King Saud University, P.O. Box 800, Riyadh 11421, Saudi Arabia; aelnour@ksu.edu.sa (A.Y.E.); AKFHK90@hotmail.com (A.A.); szahrani@ksu.edu.sa (S.M.A.-Z.); 2 Center of Excellence for Research in Engineering Materials (CEREM), King Saud University, P.O. Box 800, Riyadh 11421, Saudi Arabia; moalam@ksu.edu.sa; 3 SABIC Plastics Application Development Centre, Riyadh Techno Valley, Prince Turki Street 1, P.O. Box 5101, Riyadh 11422, Saudi Arabia; fayezaf@sabic.com (F.A.); zbashir2703@gmail.com (Z.B.)

**Keywords:** reinforced polymer composite, metal–plastics, poly(ethylene terephthalate), mechanical properties, aluminium nano-platelets

## Abstract

Previously, we reported that amorphous poly(ethylene terephthalate) (PET) filled with irregular nodular aluminium (Al) particles gave simultaneous increases in tensile modulus, tensile strength, and impact resistance, which is unusual for materials. Here, we investigated the effect of the particle shape and size by using nano-platelet Al. The Al nano-platelets had a thickness higher than graphenes and clays, but lower than mica and talc, and due to their large widths, they had high aspect ratios. Due to the ductility of Al, the platelets maintained the high aspect ratio and did not snap during injection moulding. In addition to avoiding the usual drop in tensile strength and impact, the composites with nano Al platelets gave an unusually high flexural modulus (8 GPa), which was almost double that attained practically with talc, mica, and graphene. This was because of the high tendency of the Al nano platelets to become oriented during moulding. The Al–PET composite would be a more cost-and-performance effective combination for making conductive composites. The Al is a cheaper material than graphene, surface treatment for adhesion (to PET) is unnecessary, and dispersion issues, such as exfoliation and de-aggregation, are not a problem.

## 1. Introduction

Conductive plastics combine a degree of electrical and thermal conductivity, with the easy formability of polymers. Plastic housings for electronics would be preferred over metal boxes if they can provide adequate shielding against electromagnetic interference. This can only be achieved by raising the electrical conductivity of plastics with conductive fillers. A lower level of electrical conductivity is useful for protection against static build-up and for providing gentler discharges. The demand for conductive plastics is increasing due to the miniaturisation of electronics, the advent of the electric car, and alternative energy production and storage methods. Thermally conductive plastics are sought in electronics, for example, for the lamp housings of light-emitting diodes. In the electric vehicle sector, batteries, for example, are housed in plastic trays, and their charging–discharging efficiency drops if heat is not dissipated.

Carbon black and graphite were the earliest fillers for conductive plastics and are still the commercially dominant ones. They have lower electrical and thermal conductivity than metals, but they have low density and price [1,2,3]. Often, however, high loadings are needed to achieve conductivity, and this makes the composites brittle. Since the 1970s, metal powders have been explored as conductive fillers [4]. Two types of filler distributions in the polymer matrix were distinguished for conductivity: the random distribution formed by injection-moulding, and the ‘segregated network’ that can be formed through the compression-moulding of polymers and metal powders using appropriate particle sizes [5,6]. In the random distribution, the percolation threshold where the conductivity increases by orders of magnitude is higher, typically at 20–50 vol.%, while with the segregated network, this can be achieved at ~5 vol.% of filler. In the random distribution, the high loadings that are needed lead to agglomeration and stress concentrations; in the segregated network, the mechanical continuity of the polymer matrix is reduced due to the existence of polymer–powder grain boundaries. Thus, in both approaches, three key mechanical properties (tensile strength, elongation-to-break, and impact) usually deteriorate.

In addition to the spatial distribution, the particle shape also has an influence on the critical volume fraction for increasing electrical conductivity. Conductive fibres [7] allow the electrical percolation threshold to be reached at the lowest loadings, followed by flakes, while spherical particles require the highest loadings. Commercial electrically conductive plastics based on metal fillers use steel micro-fibres [7]. Danes et al. [8] showed that, with 43.5 vol.% of 0.1-mm diameter aluminium fibres in poly(butylene terephthalate) (PBT), the thermal conductivity could be raised by up to 10×. Jankovic et al. [9] showed that copper powders with a highly dendritic morphology, when placed in poly(methyl methacrylate) (PMMA), attained the electrical percolation threshold at just 2.9 vol.%. The most desirable option is to obtain enhanced conductivity with a low conductive-filler content, thereby minimising the increase in density and the loss of mechanical properties, and allowing cost reductions.

In the last 30 years, carbon nanotubes (CNTs) [10,11,12,13,14,15] and nano platelet graphene [16,17] have been investigated as fillers in polymers, as their intrinsic thermal and electrical conductivities and mechanical properties are even higher than those of metals. However, despite many years of effort, at their current prices, CNTs and graphenes are rarely cost-effective, even at loadings of 1–5%. CNTs and graphenes are beset by two further problems: aggregation when high filler loadings (>~5 vol.%) are attempted and poor adhesion. The first problem requires complicated de-clustering processes before the introduction of the CNTs to the polymer, and the adhesion deficit requires often-impractical chemical treatments of the fillers to introduce functional groups [18,19]. The latest attempts to reduce cost involve using ‘hybrid fillers’, for example, CNTs with clays, or graphene with an inorganic mineral [20].

Hence, the metal powder route can still be a viable route for electrically and thermally conductive plastics providing a metal–plastic pair can be found where the adhesion is good. Most of the work on conductive composites using metals have employed Fe, Ni, Cu, Zn, Al, and Ag powders, and the typical plastics have been the polyolefins, poly(vinyl chloride) (PVC), poly(methyl methacrylate) (PMMa), polystyrene, styrene acrylonitrile (SAN), polyamide 6, and polycarbonate [21,22,23,24,25,26,27,28,29,30,31,32,33,34,35]. Most often, the adhesion between the metal and plastic is poor; hence, electrical conductivity may be obtained, but with a deterioration of the mechanical properties. PET has not been popular as a matrix, as it is not considered an injection-moulding plastic. This is not because PET cannot be injection-moulded, but because it crystallises too slowly. Thus, if cold moulds are used, moulded PET articles would be amorphous, or if the part is thicker than 3 mm, the article would crystallise inhomogeneously (crystalline in the core and amorphous in the skin), rendering it unusable [36]. Commercially, PBT is used as an injection-moulding polyester due to its ability to crystallise rapidly in injection-moulding time scales. However, more recent works on nanocomposites have tried PET as the matrix with clay [37], CNTs [38], and graphene [39,40]. 

Recently, Arfat et al. have investigated amorphous PET filled with nodular (irregular) Al particles, and found unusual mechanical properties that suggest that the adhesion between Al and PET is intrinsically good [41]. In order to see whether this was generally true for any Al and PET, we have investigated an Al with a defined particle shape to see the effect on properties. The particles used here were Al platelets, which were micron-sized in width and had nano thickness. In addition to the unusual combination of increased moduli, strengths, and impacts (as observed with nodular Al in PET), high flexural moduli were obtained here due to the flow-induced orientation of the Al nano platelets.

## 2. Experimental

### 2.1. Materials Used

A bottle-grade PET was obtained as semi-crystalline pellets. It had an intrinsic viscosity of 0.84 dL/g, and it had an isophthalic acid comonomer content of 2 wt.%.

Al flakes (nano platelets) from Nanografi, Germany were used as the filler. The manufacturer’s information was scant (Table 1). Hence, we characterised the powder ourselves.

### 2.2. The Particle Size Distribution of the Al Powder 

A Malvern Mastersizer 2000 (Malvern Instruments Ltd., Worcestershire, UK) was used for particle size measurement. It uses light scattering, and measures a volume-based particle size distribution (PSD). The M2000 has 52 detectors on one lens and can, thus, measure a wide range of particle sizes in one analysis without the initial fractionation of the sample.

### 2.3. Preparation of Amorphous PET Bars Filled with Al Platelets

Tensile bars of amorphous PET and composite bars with the Al powder were made by extrusion-compounding, followed by injection-moulding. The semi-crystalline PET pellets were dried in a fan oven at 150 °C overnight. The melt extrusion-compounding of Al powder with PET was completed with a table-top DSM Xplore micro-compounder (15 cm^3^) (Sittard, The Netherlands), which is a co-rotating twin-screw extruder with a melt recirculation system for multiple passes through the screw. The melt extrusion-compounding of PET pellets and aluminium powder was completed at 275 °C with a screw revolution of 100 per minute. The residence time in the extruder was 3–5 min. The compounded melt was not pelletised, but was transferred to a heated holding pot, which was an accessory of the DSM Xplore system (Micro-IM mini-injection-moulding machine). The holding pot attachment was then connected to the mini-injection-moulding machine, and the melt was injected into unheated moulds to make tensile, flexural, and Izod bars. The injection-moulding parameters for preparing the amorphous PET bars were a melt temperature of 275 °C, a mold temperature of ~25 °C, an injection pressure of 0.6 Mpa (or 6 bar), and an injection time of 45 s.

The vol.% and the corresponding wt.% loadings of the Al are shown in Table 2.

The weight of the Al powder to be added for each composition to achieve a certain volume fraction was determined from the densities of the two (ρ = 2.71 g/cm^3^ for Al and 1.333 g/cm^3^ for amorphous PET).

### 2.4. X-ray Diffractograms

The diffractograms of the Al powder, the injection-moulded PET, and the Al–PET bars were recorded to check the order in the PET. A Bruker X-ray diffractometer (D8 Discover, USA) with Cu K_α_ radiation was used and operated at 40 mA and 40 kV. The scanning speed was 2°/min, and the scanned range was 2θ = 5°–80°, with a locked scan type and 0.02° increments.

### 2.5. Thermal Analysis

Differential scanning calorimeter (DSC) analyses of amorphous PET and the Al–PET bars were carried out using a DSC-60A (Shimadzu, Tokyo, Japan) Thermal Analyser. The DSC also gives information on whether the PET phase was amorphous or crystalline. Slices cut from the PET and Al–PET bars were heated from 30 to 300 °C at 10 °C/min, held at 300 °C for 3 min to ensure sample melting, then, cooled to 30 °C at 10 °C/min. From the heating curve, the T_g_ could not be calculated due to enthalpy relaxation peaks caused by the physical ageing of the samples. The peak cold-crystallisation temperature, T_cc_, and the peak melting temperature, T_m_, were noted from the heating curves, and the enthalpies under the two peaks were measured. From the cooling curves, the peak of the crystallisation temperature from the melt, T_c_, was noted, and the T_g_ was measured.

### 2.6. Tensile Test

The tensile test was performed using a Tinius Olsen uniaxial universal testing machine (Horsham, PA, USA, Model: H100KS). The tensile bars had the following dimensions: end-to-end length L = 150 mm, width W = 12.7 mm × depth D (thickness) = 3.25 mm. Our tensile bar’s dimensions and shape corresponded, in general, to the Type I bar of ASTM D638-14, except in one detail: our bar had an end-to-end length L = 150 mm, while in the ASTM Type I bar, the end-to-end length L = 165 mm (see Supplementary Information). That is, in our bar, the grip section was shorter than in the ASTM bar by 7.5 mm, but this had no influence on the measurement, as all other features—including the gauge length—were the same (See Supplementary Information). The bar was made shorter in the grip because the mini injection-moulder had a small shot size. We, therefore, say our tensile bar was, in essence, the same as the Type I tensile bar of ASTM D638-14, and there is no effect on the validity of the modulus and strength measurements or on the conclusions drawn.

The cross-head speed used was 50 mm/min. ASTM D638-14 allows 5, 50, or 500 mm/min, and recommends the slowest according to the time to reach the extension-to-break (<5 min.). Since the pure (amorphous) PET bar took > 5 min when 5 mm/min was used, 50 mm/min was employed for the PET and the Al–PET bars. The Supplementary Information provides further details on the choice of testing speed and compliance with the ASTM standard.

### 2.7. Flexural Test

The three-point flatwise bending flexural test was performed using a Tinius Olsen uniaxial universal testing machine (Horsham, Pennsylvania, USA, Model: H100KS). The ASTM D790-03 (‘Standard Test Methods for Flexural Properties of Unreinforced and Reinforced Plastics and Electrical Insulating Materials’), Procedure A, was used to measure the flexural moduli and flexural strengths of the unfilled and filled PETs. The bar’s length was 134 mm, the width was 12.7, and the depth (thickness) was 3.25 mm. The details on our compliance with the standard are provided in the Supplementary Information.

### 2.8. Notched Izod Impact Resistance

The notched Izod impact resistance of the Al was filled and the amorphous PET bars were measured according to ASTM D-256-04, Type A, using an AMSE pendulum impact tester machine (Torino, Italy). The moulded bar had a length of 64 mm, a width of 12.7 mm, and a thickness of 3.25 mm, as set by the standard. It was notched by machine. The V-shaped notch was cut at the mid-point along the length of the bar (31.8 mm), and had full cone angle of 45 degrees and was 2.5-mm deep (radius). The distance in front of the notch in the bar was = 12.7–2.5 = 10.2 mm. The amorphous PET and all the Al–PET bars broke according to the type C break (Complete Break) mentioned in the standard, that is, upon breaking, the bar separated into two or more pieces. Hence, impact value comparisons are valid as the failure category was the same for all compositions. Further details on our compliance with the standard are provided in the Supplementary Information. 

### 2.9. Scanning Electron Microscopy (SEM)

The Al nano platelets and the Al–PET composites were examined with a scanning electron microscope (JSM-6360A, JEOL Ltd., Akishima, Japan) to see the particle shape and check on the particle size reported in the manufacturer’s data sheet.

The fractured surfaces of the amorphous PET and the Al/PET composite samples were also examined in the SEM. All samples were sputtered with a thin layer of gold and mounted on aluminium holders by using double-sided electrically conducting carbon adhesive tape.

### 2.10. Electrical Resistivity Measurement 

The resistivity measurements were made on injection-moulded plaques according to ASTM D257, using a Keithley Electrometer/High-Resistance Meter (Model 6517B) coupled with a resistivity test fixture (Model 8009). 

### 2.11. Thermal Conductivity Measurement

The thermal conductivity values were measured in triplicate using a TCi Thermal Conductivity Analyser from C-Therm Technologies (Fredericton, NB, Canada). It uses a Modified Transient Plane Source Sensor conforming to ASTM D7984 for the thermal conductivity measurements. The reported values in W/mK are an average of three different measurements through the thickness at ambient temperature. The test specimens were square plaques that were injection-moulded.

## 3. Results and Discussion

Much of the literature seeking conductive plastics focuses solely on enhanced conductivity (which is surely obtainable above a certain loading), but mechanical properties are either unreported or, where reported, show a decline. The literature on Al in various plastics also shows this trend, with some reporting the conductivity but not the accompanying mechanical properties [7,21,22,23]. However, some works on Al-filled plastics do report the mechanical properties. Bishay et al. [24] studied Al–PVC and found that the tensile strength values decreased (from a very low initial value of 3–4 MPa for PVC to under 1 MPa at 25 vol.% of Al) and the elongation-to-break decreased with increasing aluminium content. Such low strengths, coupled with low elongation-to-break, would be unsuitable for practical use, regardless of conductivity. Kakroodi et al. [25] studied malleated polyethylene (PE) filled with Al. Electrical percolation was reached at ~25 vol.%; a high notched Charpy impact resistance (non-break) was found. However, malleated PE is only a stiff rubber with a low modulus (~400 MPa), and low strength (~12 MPa with 25 vol.% Al). It is more challenging to combine good tensile strength and impact in a more rigid polymer when filled. Other works using aluminium (Al) powder in polypropylene [26] and polycarbonate [41] also reported a deterioration of tensile strength and impact resistance. Nicolais and Nicodemo reported that styrene acrylonitrile (SAN) plastic, when filled with Al and Fe powders, led to an increase in modulus but a major decrease in tensile strength and elongation-to-break [27]. The Al powder showed a bigger drop in the tensile strength, from which they inferred that the adhesion with SAN was poor [27].

In contrast, we have taken the approach to start with a metal–plastic pair which has intrinsically good adhesion, so that the mechanical properties are not impaired, knowing conductivity will follow above a certain loading. We reported that amorphous PET filled with 15 vol.% of nodular (irregular shaped) Al particles gave a raised tensile modulus of 2.1 GPa, a strength that was invariant at ~57 MPa, and an increase in the notched Izod impact resistance (from 22 J/m for amorphous PET to 51 MPa), despite the elongation-to-break dropping from 96% to 14% [41]. It is uncommon in materials to get a simultaneous increase in modulus, strength, and impact, especially when the elongation-to-break decreases. In this work, we look at Al particles with a defined shape and size (nano platelets), and show that the simultaneous increase in modulus, strength, and impact resistance is repeated as with the nodular Al, and thus, is a property of the Al–PET pair, and further, that the Al nano platelets used here lead to additional effects not observed with the nodular Al powder. To be sure of the validity of the unusual mechanical results, we have followed the ASTM standards rigorously, and subjected the results to statistical analysis where needed.

### 3.1. Particle Size Analysis of the Al Nano Platelets 

**Figure 1:**
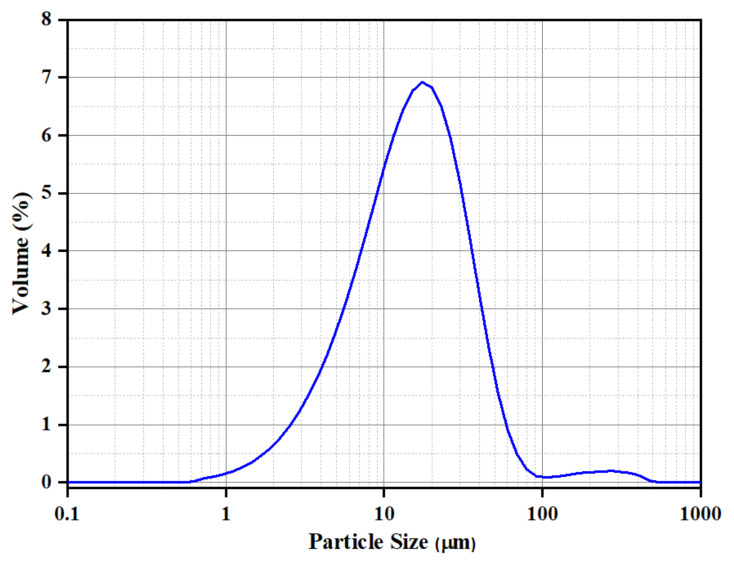
Particle size distribution of aluminium powders (Nanografi, Germany).

The particle dimensions were actually larger than specified in the manufacturer’s data sheet. Figure 1 shows the volume-based particle size distribution (PSD) of the aluminium powder. The curve shows the PSD is in the range of 1–100 μm with the peak at 11 μm (D 50 of 16 μm), and a very small fraction extending up to 500 μm. These dimensions were cross-verified by microscopy.

### 3.2. Particle Shape

The PSD in Figure 1 does not give information on the particle shape. The manufacturer gave no information of the method of manufacture of the aluminium powders. Al powders are made by gas atomisation. Depending on the atomising gas that is used to spray the molten aluminium, the particles can have an irregular/nodular shape [41], or be spherical. For the pigment and automotive paint industry, flattened flakes are made by ball milling the spherical or the nodular powder. 

**Figure 2:**
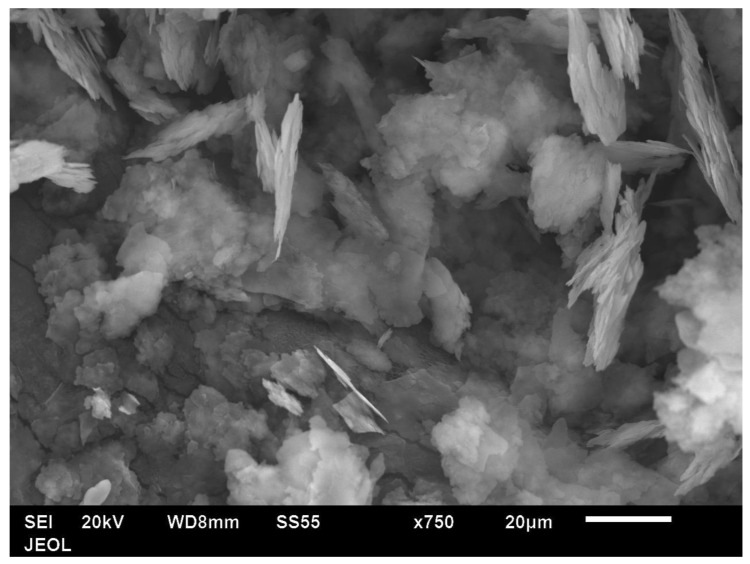
Low-magnification SEM image of Al powder (Nanografi, Germany) shows a platy material with few flakes lying edgewise. However, due to clumping, the platelet thickness cannot be estimated accurately.

**Figure 3:**
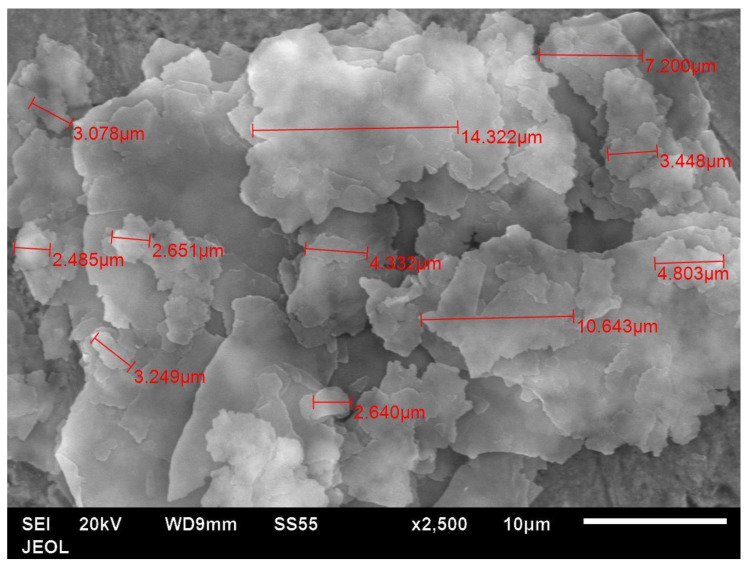
SEM image of Al 1 powder (Nanografi, Germany) shows flakes and their in-plane dimensions. The shapes are not polygonal, and they have rounded edges.

The particle shape of the aluminium Al flakes is depicted in Figure 2 and Figure 3. Unlike platelet minerals such as mica, the Al platelets did not have any recognisable regular polygonal shapes (rectangular, circular, hexagonal, square, rhombus, etc.). The width of the platelets was in the range of about 2–15 μm, with an average of ~5 μm. The Al platelets had an irregular contour (Figure 3), with rounded edges, instead of the sharp corners found with mineral platelets. The lack of sharp edges is beneficial, as it reduces stress concentrations. The manufacturer’s data sheet did not indicate the thickness, and it could not be deduced from the laser light-scattering measurement for particle size in Figure 1. It was difficult to estimate the thickness directly from the powder, as most flakes lay flat, and edge-on dispositions (Figure 2) showed clumping. The platelet thickness could be measured more accurately from the flakes embedded in the PET composite (to be shown later). The thickness of the platelets (obtained from measurements on the composites) lay in the range of 50 to 80 nm. The manufacturer has labelled this as a micro-Al powder; however, since one dimension is in the low-nm range, these Al platelets can be classified as a nano material. They are closer to nano platelet materials such clays and graphene than they are to mica and talc. 

For mica and talc, the plate thickness is about 1 μm. In other nano platelet materials such as clay and graphene, the thickness may be in nms, and the width may be in several tens or hundreds of nms. However, in Nanografi’s Al flakes, the thickness is in several tens of nms, but the width is quite large (several microns). Hence, the aspect ratio is still high, as with graphene and clay. For example, from the microscope, the average width was ~5 μm, and the average thickness was ~65 nm; hence, the average aspect ratio was ~77.

### 3.3. The Order in the PET Phase after Injection-Moulding, and the Appearance of the Composites

PET articles can be obtained in amorphous or semi-crystalline form, depending on the cooling rate, and it is important to ascertain this as it affects thermomechanical properties. This aspect is often unspecified or unrecognised in many works using PET as a matrix with clay, graphene, and CNTs. 

**Figure 4:**
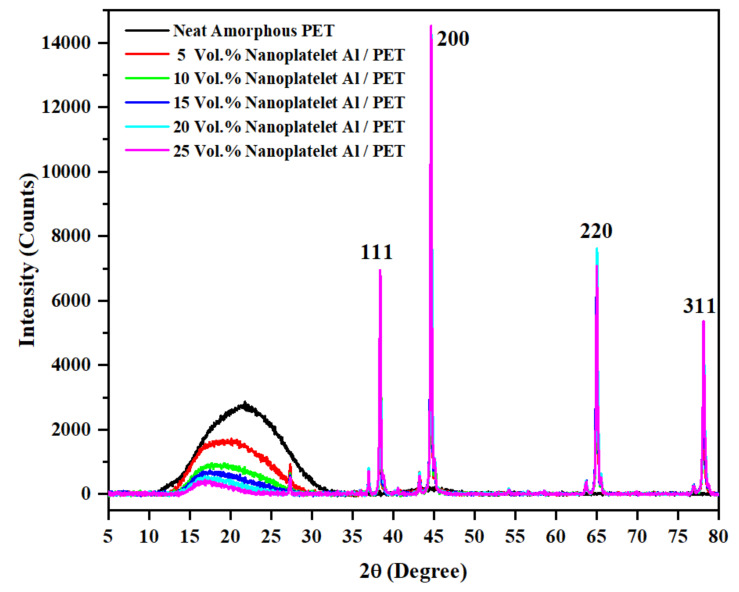
Wide-angle X-ray diffractogram of the nano platelet Al–PET composites. The broad peak at 2θ values between 11° and 33°confirms that the PET is in an amorphous state in the bars. The tall sharp peaks are from Al and the Miller indices are indicated. The small sharp peaks at 2θ ~27°, 37°, 44°, 64°, and 77° are artefacts.

An X-ray diffractogram and a DSC thermogram of the white PET pellets confirmed it was semi-crystalline (See Figures S1 and S2 in the Supplementary Information). The pure PET bar moulded with cold moulds, on the other hand, was transparent, and this was amorphous (see black curve in Figure 4). Due to the opacity in the Al-filled PET, visually, one could not gauge whether the PET portion was amorphous. To avoid any ambiguity on the state of the order in the PET phase, X-ray diffractograms of the PET bar and the Al–PET bars were recorded. These displayed a broad peak at 2θ values between 11° and 33° (Figure 4), indicating the PET in the composites was also in the amorphous phase. There were also superimposed sharp peaks in Figure 4 from the aluminium embedded in the amorphous PET, with 2θ values of 38.43°, 44.68°, 65.04°, and 78.16°, and the standard Miller indices corresponding to face-centred cubic Al are shown. The X-ray diffractogram of the pure aluminium nano-platelet powder is given in the Supplementary Information (Figure S3); it showed sharp peaks at 2θ values of 38.36°, 44.60°, 64.94°, and 78.06°, similar to Figure 4, and similar to what is reported in the literature [42] for ultrafine Al powder (2θ = 38.5°, 44.8°, 65.2°and 78.3°, [42]). Note that there are a number of small sharp peaks in Figure 4 at 2θ ~27°, 37°, 44°, 64°, and 77°. These are not from the Al or the PET, but are artefacts (stray reflexions) because these small peaks in Figure 4 did not appear in the X-ray diffractograms of the Al powder or the pure PET pellets (see Supplementary Figures S1 and S3 respectively). The Al powder and the PET pellets were placed in a different sample holder from that used for the bars.

**Figure 5:**
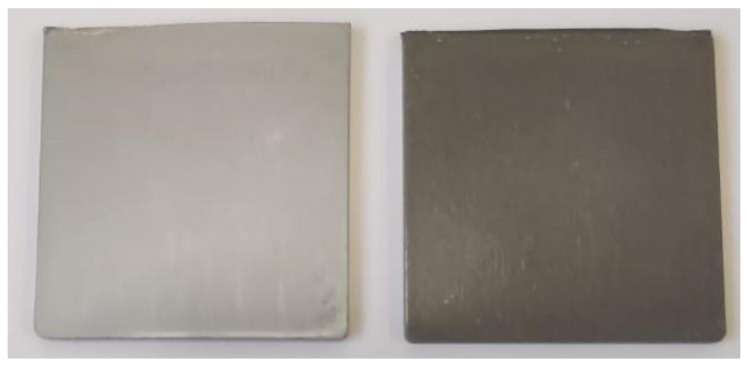
(**Left**): Plaque of amorphous PET with Al nano platelets is silvery. (**Right**): Plaque of amorphous PET with nodular Al particles is dark grey. The appearance is directly related to the particle shape.

One effect of the Al particle’s shape was in the appearance of the moulded bars and plaques. Bars and plaques of amorphous PET moulded with Al platelets here were silvery, unlike those made with irregular, nodular Al in the previous work [41], which were dark grey (Figure 5). The silvery appearance of the Al platelets was due to them orienting parallel to the surface of the bars and plaques, giving enhanced specular reflection (the orientation tendency will be proved by microscopy).

### 3.4. The Nucleating Effect of Al Particles on the T_g_, the Cold Crystallisation, and Crystallisation from the Melt

**Figure 6:**
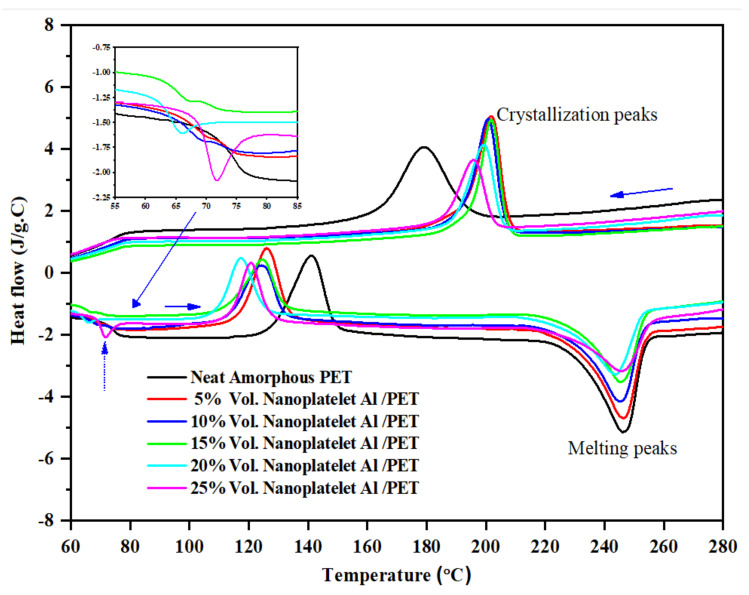
First DSC cycle curves at 10 °C/min. The bottom curve is for heating of the amorphous PET and the Al platelet/amorphous PET composites. The inset (arrow) shows expansion of the enthalpy relaxation peak at the T_g_, seen in the heating curves (vertical arrow), due to physical ageing of the samples. The T_g_ was, therefore, measured from the cooling curves (top).

The DSC thermograms (first heating and first cooling after melting), of the amorphous PET and Al–PET bars were collected. These are shown in Figure 6, and the calorimetric data from them are collected in Table 3. 

The following are the key observations: (1) the heating curves were typical of amorphous PET, thus confirming the PET in Al–PETs were in the amorphous phase, as also shown directly by the X-ray diffractograms in Figure 4; (2) there was a shift in cold crystallisation peak T_cc_ to lower values in the Al–PETs compared with the amorphous PET; (3) the melt crystallisation peak T_c_ showed a shift in the Al–PETs to higher temperatures. 

Effects (2) and (3) indicate that the Al nano platelets act as nucleating agents for the crystallisation of PET from the glassy state and also from the melt. This was observed also with the nodular (irregular) Al particles previously, and hence, in this regard, the Al particle’s shape shows no major effect [41]. Other fillers also show similar nucleating effects.

It would have been desirable if the PET could crystallise as fast as PBT. However, despite the nucleating effect of Al, the PET still did not crystallise as fast as PBT and the matrix in the Al–PET composite bars was amorphous. The crystal growth rate of the PET after crystal nucleation is not greatly altered.

In Table 3, the filler content is in volume %. T_cc_ is the cold crystallisation temperature from the rubbery state after crossing the T_g_ when the bar was heated, T_m_ is the melting peak, T_c_ is the crystallisation peak after cooling from the melt, **∆**H_cc_ is the enthalpy of cold crystallisation, and **∆**H_c_ is the enthalpy for crystallisation from the melt. Due to physical ageing, there were enthalpy relaxation peaks obscuring the glass transition temperature T_g_ in the heating curve (see upward arrow and the inset in Figure 6). The T_g_ was, therefore, measured from the cooling curve in Figure 6.

### 3.5. Tensile Properties of Amorphous PET with Al Platelets 

**Figure 7:**
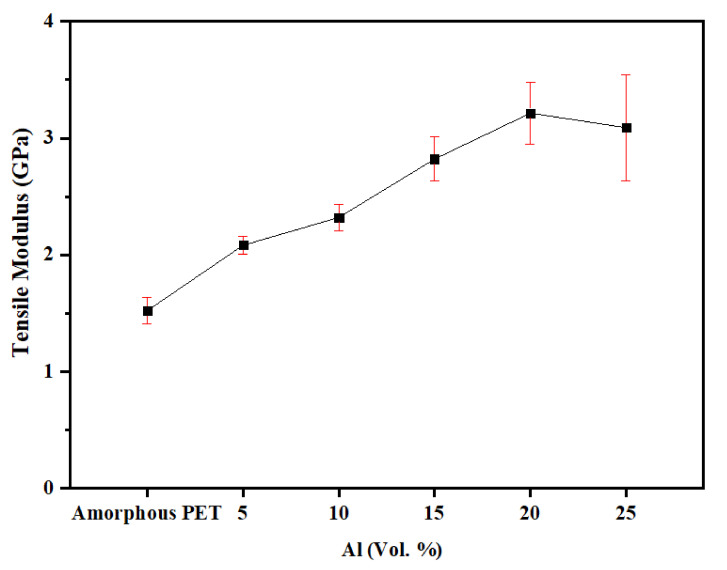
Tensile modulus of amorphous PET filled with Al nano platelets.

**Figure 8:**
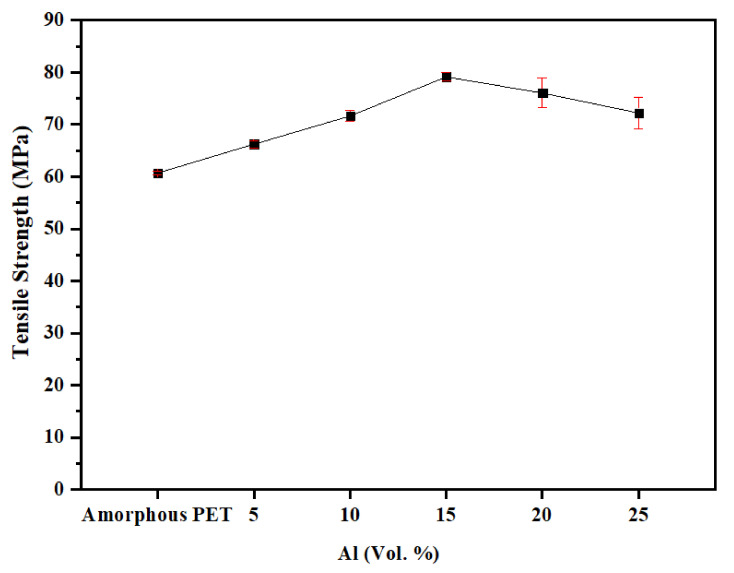
Tensile strength of amorphous PET filled with Al nano platelets.

**Figure 9:**
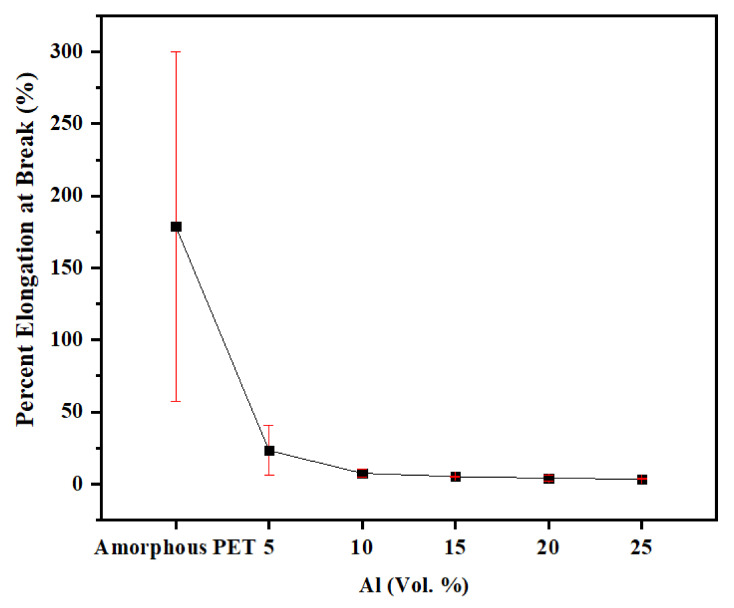
Elongation-to-break of amorphous PET filled with Al nano platelets.

The values of the tensile modulus and tensile strength, and the elongation-to-break of the Al platelet–PET composites, as a function of Al loading levels, are shown in Figure 7, Figure 8, and Figure 9, respectively. The tensile modulus increased continuously with an increase in Al loading, and the variation was low. At 25 vol.% of Al, the modulus had doubled (Figure 7). This level of increase is generally observed when any rigid filler added to a rigid plastic matrix.

The tensile strength of the unfilled amorphous PET was 60.7 MPa (Figure 8). We are aligned with the literature values (56 MPa in Aravinthan and Kale [43] and a little under 60 MPa in Novello et al. [44]), and differences in testing rate and the PET’s I.V. do not make much difference. Figure 8 shows that the tensile strengths of PETs containing the Al nano platelets were higher than the amorphous PET. The tensile strength increased from 60.7 MPa to 79 MPa at 15 vol.% of Al flakes. The tensile strength appeared to reach a maximum and levelled off beyond 20 vol.% of Al, but it did not drop below the base polymer. The standard deviation for the tensile strength was low; hence, one can conclude that the addition of Al nano platelets does not cause a drop in strength, but, in fact, increases it. This is unusual, as in most cases of filler composites, the strength decreases with filler content [26,27,45,46]. 

Shabafrooz et al. [39] describe graphene nano platelet-PET composites, compounded and injection-moulded with the same equipment we have used. Shabafrooz’s X-ray pattern showed the PET in their graphene-nano platelet composites was amorphous, and hence, we can compare it with our Al–amorphous PET composites. Despite their higher aspect ratio, with 10 wt.% graphene, a tensile modulus of ~2.9 GPa and a strength of ~57 MPa was obtained, compared with 1.8 GPa and 54 MPa for their unfilled PET. The level of reinforcement achieved with graphene is not commensurate with its current price, even at the lower amounts used. The only advantage over Al is that graphene scores lower on density.

The tensile elongation-to-break of the Al composites dropped exponentially from 179% for amorphous PET to 2–3% in the composites (see Figure 9; see also the stress–strain curves in Figures S4–S9 in the Supplementary Material). This is always observed in filled composites and it is generally attributed to matrix immobilisation by the filler [26,27,46]. Note that the average elongation-to-break for amorphous PET (179%) was obtained with a cross-head speed of 50 mm/min, and it can be higher or lower than this depending on testing speed, storage time, and molecular weight. It shows higher variability after prolonged storage (see stress–strain curves in Figure S4, Supplementary Information). With 2 mm/min, and freshly moulded amorphous PET, the elongation-to-break of amorphous PET would be ~550%. The elongation-to-break for amorphous PET can be reduced due to physical ageing caused by prolonged storage; thus, in a previous work, the elongation-to-break of amorphous PET was 96% at a cross-head speed of 50 mm/min [41]. This is because PET necks and draws even at room temperature. The relative drop in elongation-to-break was in fact more severe in Al–PET than in other high T_g_ plastics with filler particles, where the unfilled polymer had a lower elongation, of typically 20–50%. For example, in a poly(phenylene oxide)–high impact polystyrene blend, the elongation-to-break was 20% for the unfilled polymer [47], but when filled with 25 wt.% mica, it dropped to ~4%. In an ABS copolymer, the elongation-to-break was 30–41%, and this dropped to ~5% when filled with 1 vol.% of steel fibres [8]. The drop in elongation-to-break is often correlated with a decrease in toughness. The worst combination is lowered strength and decreased elongation-to-break, as it signifies a weak and brittle material. As will be shown, despite the bigger drop in elongation-to-break (in both relative and absolute terms) in the Al–PET composites, compared with other thermoplastics with rigid fillers, the impact resistance did not decrease.

### 3.6. Flexural Properties of Amorphous PET Composites with Al Nano Platelets

**Figure 10:**
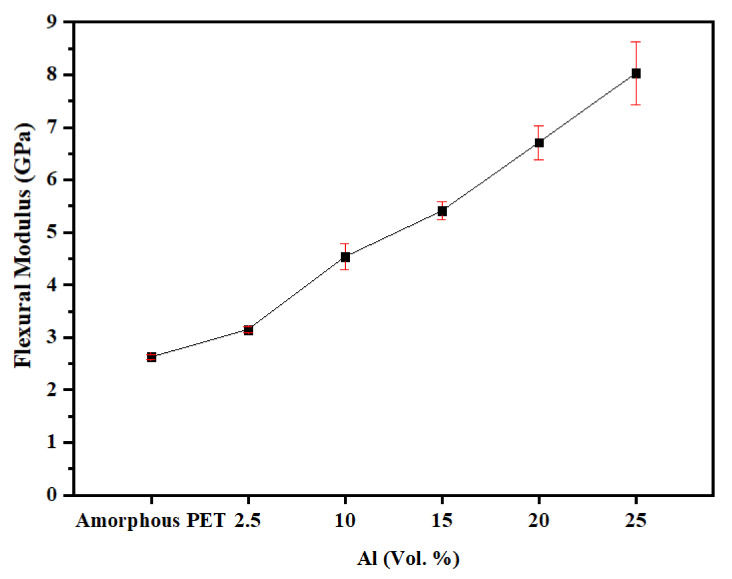
Flexural modulus of amorphous PET filled with Al nano platelets.

Figure 10 shows that the flexural modulus increased and reached a value of 8 GPa at 25 vol.% of Al platelets. With nodular (irregular) Al particles in PET, the highest flexural modulus reached was 3.24 GPa at 15 vol.% [41]; with the Al nano platelets here, at 15 vol.%, the value was 5.41 GPa. Polymers filled with 40 vol.% of short glass fibres reached flexural moduli of 9–10 GPa. The tendency for orientation of the nano platelets parallel to the largest surface of the bar (shown later in the SEM pictures) no doubt contributes to the increased flexural modulus. We could not find a parallel in the literature of platelet-composites where values of flexural moduli as high as 8 GPa were obtained. Table 4 collects some literature data on PETs filled with other platelet fillers (talc, nano mica, graphene and graphene oxide, calcium terephthalate). It can be seen from Table 4 that the combination of increased tensile modulus with increased tensile strength, increased flexural modulus, increased flexural strength, and increased impact resistance is only obtained with Al–PET. With graphenes and clays, higher loadings than 5–10% do not appear to be feasible due to the rise in viscosity, and the loadings in the 1–10% range bring a modest increase in some mechanical properties but at the expense of others, and the cost also makes it impractical. Shim et al.’s work (Table 4) involved taking graphene oxide (itself made from graphene), functionalising the graphene oxide further, and then solution-blending it with PET at very low dilutions. They [48] showed an increase in modulus and strength with addition of 0.5 wt.%, and even an increase in the elongation-to-break of the composite, but puzzlingly, their base PET had the lowest values of modulus and strength in the literature (modulus of 0.49 GPa for PET, whereas most authors show a value of 1.5–2.9 Gpa and a strength of 32 MPa, instead of ~60 MPa shown by all others, Table 4). After incorporating functionalised graphene oxide by solution-blending [48], Shim’s highest modulus was only 0.79 GPa (that is, below the value of unfilled PET found by all others) and the highest strength was 60 MPa (that is, the same as unfilled PET found by others, Table 4).

Similar trends were observed with platelet fillers embedded in other polymers. Asyadi et al. [53] studied mica-filled 70/30 polycarbonate/(acrylonitrile-butadiene-styrene) (70/30 PC/ABS) blends. The flexural modulus of the unfilled blend was 2.1 GPa and the highest obtained was 2.2 GPa, with a 30 wt.% mica-filled blend. Both treated and untreated mica were tried, but it did not make much difference. In Sahai and Pawar’s [47] study of mica in the high-T_g_ polymer ‘Noryl’ (an amorphous polyphenylene oxide (PPO)-high-impact polystyrene blend), the flexural modulus of the mica-Noryl composite increased from 2.6 GPa for the unfilled polymer blend to 4.1 GPa at 25 wt.% of mica.

It is remarkable that, although Al has a tensile modulus of 70 GPa, which is lower than for minerals (muscovite mica’s modulus is 172 GPa) and also much lower than clays and graphenes, a higher flexural modulus of 8 GPa was reached (Figure 10) in the Al nano platelet–PET composites. This is because of the high degree of Al platelet orientation achieved compared with the other platelet composites, and this, in turn, occurred due to the high aspect ratio of the Al nano platelets we used, and their tendency to not break in the extrusion.

**Figure 11:**
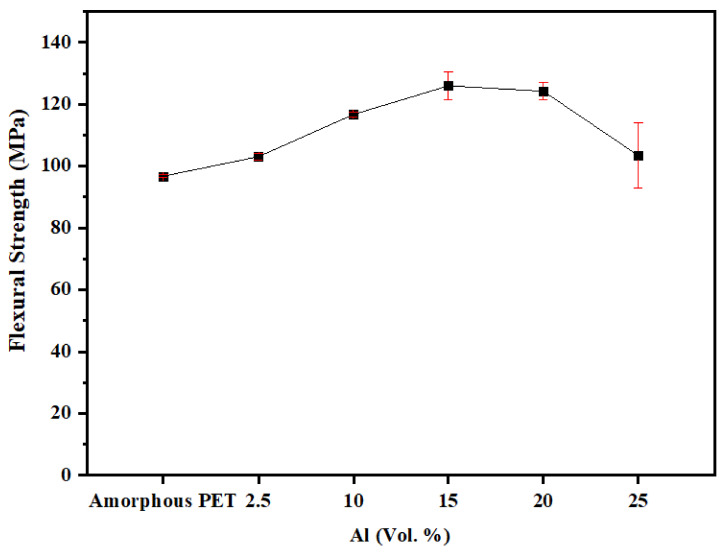
Flexural strength of amorphous PET filled with Al nano platelets.

Figure 11 indicates that the flexural strengths for the amorphous PET composites with Al nano platelets lay in the range of 100–120 Mpa. Generally, they were higher or were comparable to the base amorphous PET (flexural strength of 96.7 Mpa). At some loadings of Al platelets (15 and 20 vol.%), there was an increase of ~25% in the flexural strength. The variations in both the flexural moduli and flexural strengths were low, except for the highest loading, where aggregation might affect the result.

### 3.7. Impact Resistance of Amorphous PET Composites with Al Nano Platelets 

PET has a high impact resistance when un-notched, but notching reduces this and makes it brittle [54]. Thus, PET is at the lower end of the scale for impact resistance amongst polymers (notched Izod impact strength < 50 J/m). Only one high-T_g_ polymer, polycarbonate, has a super-high impact resistance of ~1000 J/m without any added impact modifiers. The impact resistance is most important and usually is the let-down in most rigid-filler composites. When a material is already at the lower end of the impact scale, it is imperative not to compromise this property, even if some other functionality such as conductivity is gained. Impact toughening usually involves the addition of rubber particles, but this compromises the modulus and strength.

**Figure 12:**
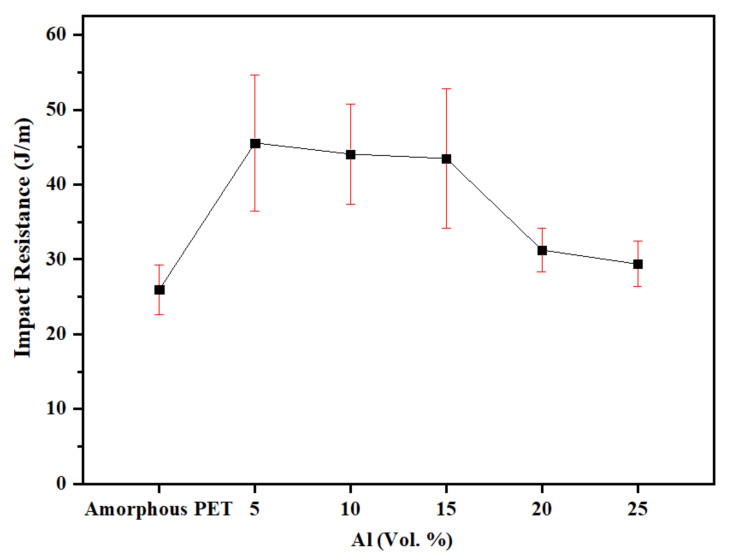
Notched Izod impact resistance of amorphous PET filled with Al nano platelets.

The comparison of the notched Izod impact resistance of amorphous PET with and without platelet Al as a function of Al loading levels is shown in Figure 12. The notched Izod resistance value we found for the amorphous PET was 25.9 J/m. Aravinthan and Kale [43] reported a value of 25 J/m. 

Figure 12 shows that the addition of the Al platelets increased the impact resistance above that of the amorphous PET. As the impact had higher variability than the other mechanical properties, we applied a statistical significance test (*t* test) to check if the differences indicated different populations of impact resistance (Table 5). According to the significance test, all the Al–PET compositions were significantly different (higher) from the unfilled amorphous PET (25.9 J/m). The impact resistance reached a plateau value of 45 J/m between 5–15 vol.% of Al, and then decreased, but in all cases, it did not drop below that of the matrix. This could be seen also in the minimum, median, and maximum values of the impact resistance (not shown), which were higher for all the Al-filled PETs than the unfilled PET. The test (Table 5) and Figure 12 indicated that there was no significant difference between the PETs with 5, 10, and 15 vol.% of Al platelets.

It is well known that tensile modulus and impact resistance go in opposite directions in filler composites where the matrix and the filler are rigid [45]. With untreated clay [50] and graphene [39] in amorphous PET, the impact resistance decreased well below that of the unfilled plastic with an increase of filler loading. A similar trend is seen with mica in polyphenylene oxide [47], mica in PBT [55], and talc in PP [56], and in a conductive plastic of acrylonitrile-butadiene-styrene (ABS) filled with 1–5 vol.% of chopped steel fibres [7].

The literature on carbon nanotube (CNT)–polymer composites, especially with untreated CNTs, also shows an increase in tensile modulus but a degradation of the tensile strength [57]; the extension-to-break always drops and, most often, with it drops the impact resistance. Al-Saleh [12] reported an unusual case of a PP filled with an untreated CNT where an increase in tensile strength was obtained—but the toughness dropped in a linear manner. Thus, despite the intrinsic conductivity of CNTs and graphene, in practice, it is difficult to incorporate them at loadings with a marked increase in conductivity without a drop in mechanical properties. It is noted that rigid mineral particles have, in a few cases, increased toughness and strength. Examples are nano calcium carbonate in polyethylene [58], and nano TiO_2_ in epoxy, but these are exceptions [45]. The Al–PET combination belongs to the exception list.

### 3.8. Fracture Morphology of Nano Platelet Al–PET Composites 

**Figure 13:**
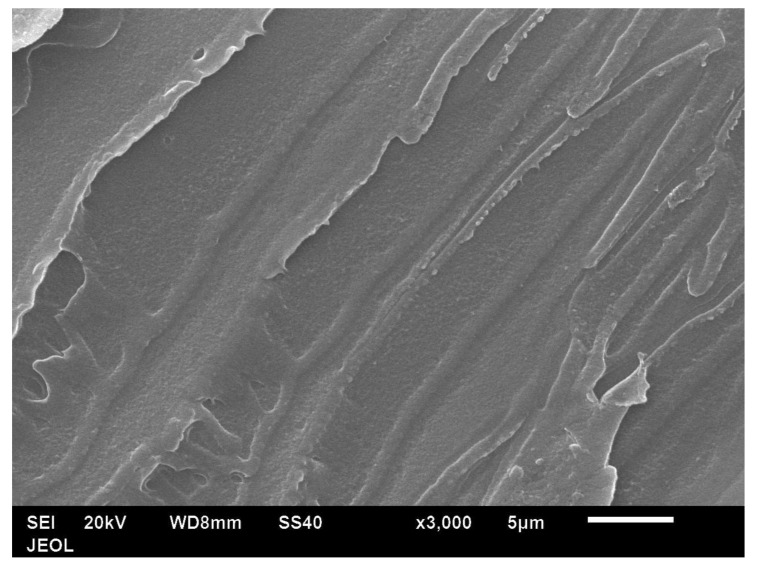
Fracture surface of the amorphous PET.

**Figure 14:**
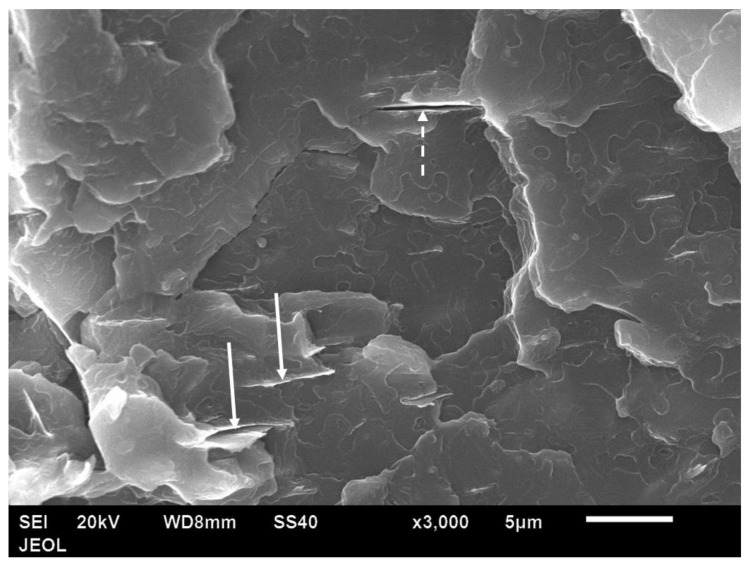
Fracture surface of 5 vol.% Al nano platelet–amorphous PET bar. Platelets were observed only edgewise. Downwards arrows indicate Al flakes with PET adhered to them. Upwards dotted arrow indicates a platelet with debonding on one face.

**Figure 15:**
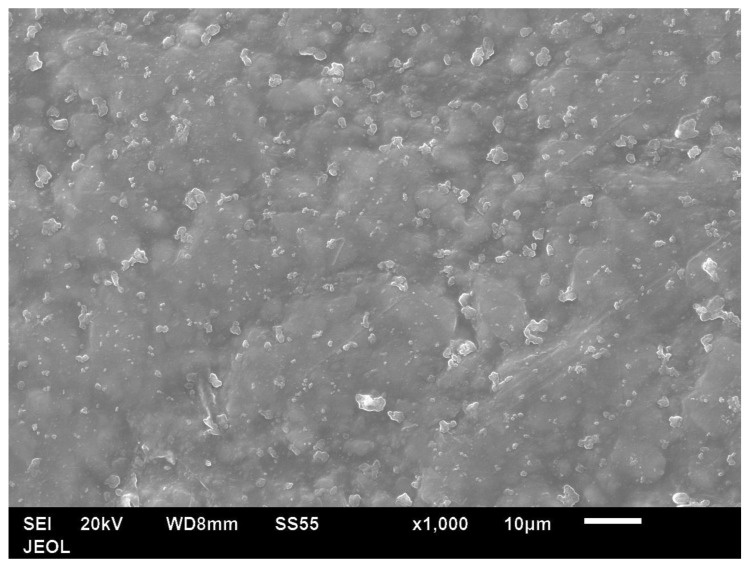
Low-magnification plan view of largest surface of the unfractured bar with 5 vol.% Al platelet–amorphous PET. Al platelets show a mostly planar orientation with respect to the principal face of the bar.

**Figure 16:**
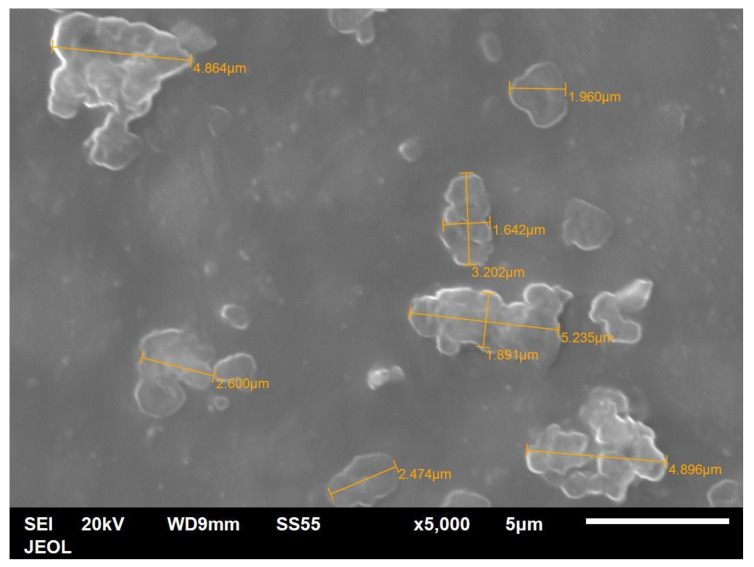
Higher-magnification view of the principal face of the unfractured PET bar with 5 vol.% Al. The Al platelets lie flat and not edgewise. Note the flakes do not have sharp edges, they have rounded corners.

Figure 13 shows the fracture surfaces of amorphous PET. Figure 14 shows the bar with 5 vol.% Al; even at the lowest loading, the platelets were seen only edge-wise (and never face-wise) in the bar’s fracture surface. The platelets were spaced 5–10 μm apart. Most of the platelets showed a coating of PET, indicating natural adhesion. Rarely, a platelet showed debonding on one face (Figure 14). Figure 15 shows the plan view of the unfractured principal surface of the Izod bar. The platelets were observed only in planar view and not in edge-on view. Figure 14 and Figure 15 suggest that the flow during the filling of the bar cavity leads to simple parallel orientation; that is, the platelets lie parallel to the widest surface of the Izod bar (width = 12.8 mm) and are oriented edge-on in the bar’s transverse cross section. In contrast, Farhoodi’s pictures of fractured cross-sections of PET with 1% and 3% mica nano platelets show platelets edge-on (~0.5 μm in width, ~50–100-nm thick), but with random orientation [51]. Figure 16 shows a higher-magnification view of the flakes on the largest surface of the bar. There is no sign of breakage due to the flow.

**Figure 17:**
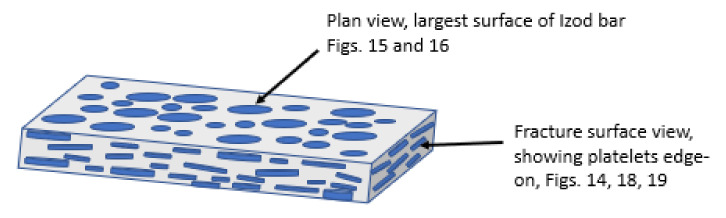
Schematic of orientation of Al platelets in injection-moulded Izod and flexural bars, as deduced from the SEM pictures of the fracture surface (horizontal arrow) corresponding to Figure 14, and also Figure 18 and Figure 19, and the plan view of the bar (top arrow), whose SEM pictures are in Figure 15 and Figure 16. The platelets are sketched, for simplicity, as disks (not to scale), although Figure 3 and Figure 16 show they are, in fact, irregular.

Figure 17 shows a schematic diagram of the flow orientation of the Al platelets in the injection moulded bar, based on the SEM pictures in Figure 14, Figure 15 and Figure 16. Shabafrooz et al. [39] deduced, by X-ray diffraction, a similar orientation of graphene platelets in injection-moulded PET bars.

**Figure 18:**
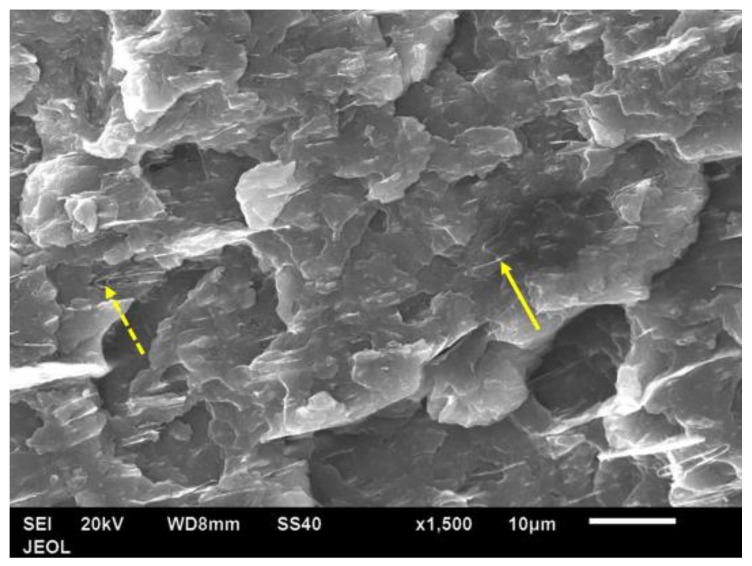
SEM image of a fracture cross-section of a 15 vol.% Al platelet–amorphous PET composite. The platelets are seen only edge-on (arrow). The dotted arrow shows two platelets that have folded back on themselves.

Figure 18 shows the fracture cross-section of a bar with 15 vol.% of Al platelets. Again, the platelets were seen edge-wise only, and as the number of platelets increased, there was a high degree of parallelism, and the inter-platelet distance reduced to ~2 μm. The dotted arrow in Figure 18 shows two platelets which, remarkably, folded back on themselves due to their excessive width—this shows the ductility of the Al. In a more brittle platelet material such as mica or talc, such a feature is unseen, as the platelet would snap. Very few pull-outs and slot holes were seen (Figure 18), and there were usually no gaps between the platelets and matrix, which confirms that the adhesion is naturally good. In contrast, in Osman and Mariatti’s work using Al flakes in PP, pull-outs are seen in the fracture section [26].

**Figure 19:**
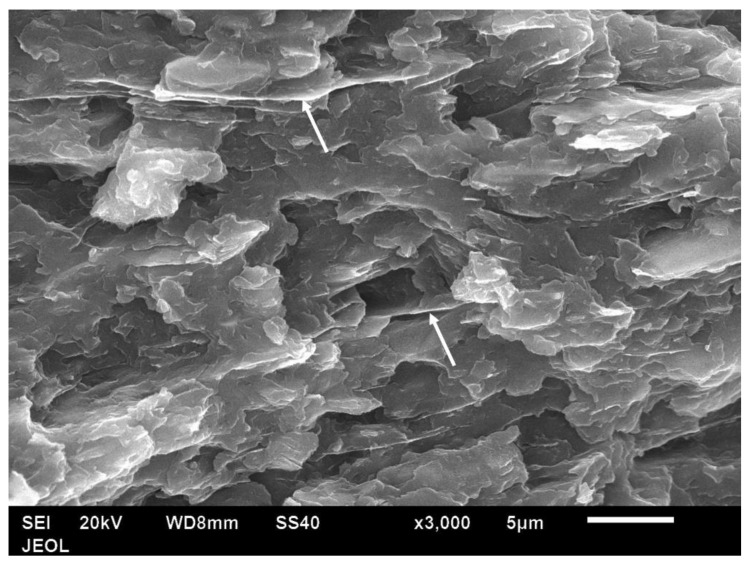
Fracture surface of 25 vol.% Al platelet–amorphous PET. Al flakes are seen only edgewise (arrow). Arrows show a protruding flake covered with PET.

**Figure 20:**
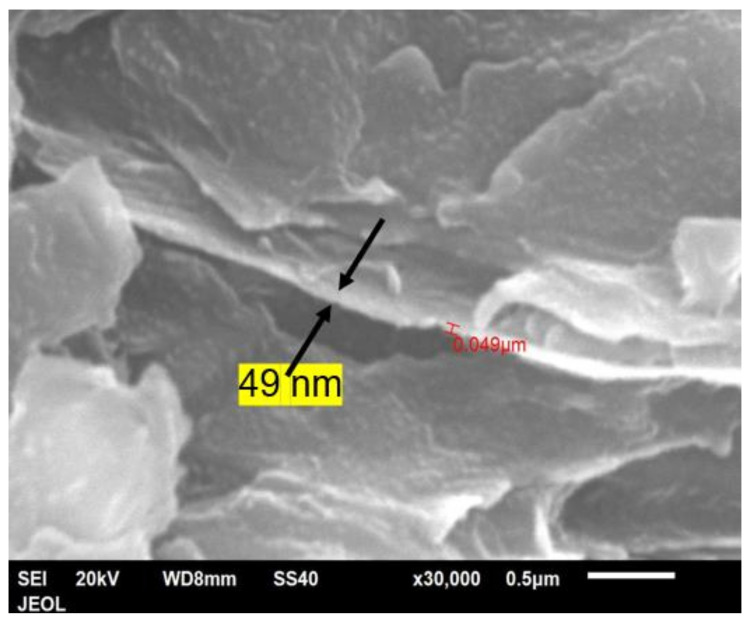
High-magnification SEM image of the 25 vol.% Al platelet–PET composite; the Al platelet is 49-nm thick and about 2-μm wide. The aspect ratio is ~40.

Figure 19 shows the fracture cross-section of a bar with 25 vol.% of Al platelets. Again, the platelets were seen only edgewise and are parallel to each other, and the distance between them is reduced to 1 μm or lower. Where some platelets are overhanging or protruding, one can see there is a coating of polymer on them. As mentioned, the thickness of the platelets could not be measured accurately from the powder due to some clumping (Figure 2). However, expanded views of the Al platelets in broken bars (Figure 20) indicated a thickness range between 0.05–0.08 μm, or 50–80 nm. The thickness of the Al platelets is, thus, in the range of tens of nm, compared with mica, where the thicknesses appear to be in the range of 500–1000 nm [53,55].

The high degree-of-orientation and parallelism between the platelets is a result of the self-ordering tendency arising from the large aspect ratio and the high flow orientation. These would explain the highest flexural modulus of 8 GPa that was attained with the 25 vol.% (Figure 10). The aspect ratio was not degraded much due to the ductility of the Al, which made the platelets fold rather than snap. Thus, PET filled with nano-flake Al would be useful in thin-wall mouldings, for example, the casing of a mobile phone. The orientation of the Al platelets would give high flexural moduli, and the silvery appearance, combined with the natural gloss of PET, would give an attractive appearance. Aluminium flakes are used to give a metallised appearance in automotive plastics, but it is noted that gating must be such that impinging flow fronts are avoided to prevent weld lines, where the platelets would be oriented normal to the surface, and would, thus, appear dark [59].

### 3.9. Properties of Aluminium and Adhesion between Al and PET

We need to consider how it is that one can get an increase in modulus, tensile strength, and impact resistance—despite a drop in elongation-to-break that was even more drastic than with other filled plastics.

Pure aluminium has a combination of interesting mechanical properties with an intermediate–high modulus of 70 GPa, a strength of 90 MPa, and an elongation-to-break of ~45% [60,61]. The modulus and strength are low for metals in absolute terms, yet aluminium’s density is low (2.7 g/cm^3^) amongst metals, so the specific modulus becomes comparable to that of steel. The low strength of pure aluminium means it is not used for structural purposes; however, by alloying with other elements, it becomes a structural metal: the strength can be increased to 300–600 MPa, while the elongation decreases to ~28%. 

A close equivalence is with E-Glass, which has a modulus of 72 GPa, a tensile strength of 3.45 to 3.79 GPa, but with a lower elongation-to-break of 4.8%, and a density of 2. 6 g/cm^3^. The pure Al has similar modulus and density as E-glass fibre, but its strength is lower than glass, and, in fact, is similar to that of a polymer; however, it has a high elongation-to-break compared with glass. The high elongation-to-break means that Al particles are ductile and less likely to break than glass fibres, due to higher stresses during injection-moulding.

It follows that, if Al is incorporated into a thermoplastic matrix, it would give an interesting combination of properties—provided the adhesion is good. It would be a sort of rubber-toughener that does not cause a drop in modulus. From the mechanical properties, one has to infer that the adhesion between Al and amorphous PET is good. If not, one would see a drop in tensile strength and impact resistance—as reported with Al in PP [26], or Al in SAN [27].

The rarity of pull-outs and dropouts in the fractured cross-section for the platelets embedded in PET, in contrast to what is seen in Al-PP [26], is direct evidence of good adhesion. We add the following, easily verifiable experimental evidence we have provided to prove that the adhesion between Al and PET can be strong. If PET is compression-moulded to form a film between aluminium foil, the foil is not peelable from the PET film. It is even difficult to scrape the Al foil off the PET film with a sharp knife. In contrast, if PE, PP, or PC are compression-moulded with aluminium foil as a backing, the metal foil can be peeled off the plastic film completely. 

On the mechanism of the adhesion, we believe that it is not directly between PET and the aluminium. Any aluminium surface reacts spontaneously with oxygen and water and forms a coating which is a mixture of aluminium oxide and hydroxide. As we did not take any steps to remove the coating layer, it must be present with the Al platelets. The outer surface of the oxide (Al_2_O_3_) layer is hydroxylated with OH groups. The oxide helps to prevent corrosion of the metal while the hydroxide groups are useful for the adhesion of paint and lacquers. J. van den Brand et al. [62] have used infrared reflection spectroscopy to study the adhesion of molecular ester compounds (dimethyl adipate) with aluminium. They showed that the C=O of the ester hydrogen-bonds with the -OH groups of the oxide layer on the aluminium. We believe this is also the mechanism by which a polyester such as PET bonds strongly with aluminium. In glass fibre composites, the hydroxylation of Si–O–Si to Si–OH provides the scope for bonding to polymers with polar groups, but silane coupling agents are used to enhance it. This appears to be unnecessary with Al and PET.

A particularly interesting contrast with PET filled with Al nano platelets is PET filled with calcium terephthalate platelets (Table 4). Dominici et al. [52] showed that calcium terephthalate forms nano platelets that were about 5μm wide and 50 ± 5 nm-thick—that is, similar dimensions and aspect ratios as the Al platelets here. However, the platelets were square or rectangular in shape, and had sharp corners. Further, Dominici et al. [52] indicated that a high degree of immobilisation of the PET chains occurs, leading to a substantial ‘rigid amorphous fraction’. This was attributed to π–π interactions between the benzene rings of the calcium terephthalate and the PET. The benzene rings of the calcium salt and the polyester could lie parallel. This led to a composite containing a rigid but brittle ionic filler, with strong bonding to the PET matrix. The result was that a high tensile modulus of ~5 GPa could be obtained, but the tensile and flexural strength decreased greatly after 3 wt.% (Table 4), unlike the Al–PET. Dominici et al. stated [52] that when the loading was too high (2–20 wt.%), the PET nano-composites with calcium terephthalate became ‘extremely fragile’.

In summary, if the adhesion is weak or non-existent, as with Al–PP, then pull-outs of the Al platelets will occur and the tensile strength will drop, as seen in the work of Osman and Mariatti [26]. If the filler is rigid and strong but brittle, and the adhesion to the matrix is too strong (as with calcium terephthalate + PET) [52], then the nano composite will show increased tensile and flexural moduli at very low filler content, but the strength and impact will be poor if the filler loading is raised beyond 2%. The combination of high matrix immobilisation and strong adhesion to a brittle filler, evidently, is problematic [52]. When the filler is rigid but ductile as it is with Al, and the adhesion of the matrix is of an intermediate strength as it is with PET, then it is possible to beget a simultaneous increase in modulus, strength, and impact resistance—despite matrix immobilization and decreases in elongation-to-break.

### 3.10. Electrical and Thermal Conductivity 

The electrical conductivity of the amorphous PET was 10^−13^ S/cm. The electrical conductivity of even the bar with the highest loading (25 vol% of Al) did not show a sharp upshoot, and its value was 10^−12^ S/cm. Figure 18 shows that while the Al nano platelets are highly oriented, almost none are in contact with each other to create the continuity for conduction. We have attained a system where the conductive filler had not degraded the mechanical properties of the PET, but we did not quite reach the percolation threshold for electrical conductivity at 25 vol.%, although we believe we are close to it. It was not possible to extrude with Al platelet content >25 vol.% due to the rise in melt viscosity. We have managed to breach the percolation threshold for Al–PET with an alternative processing method we developed, called hot powder compaction, which allows for higher loading of the Al (to be published). Further, Al microfibres should allow connectivity at lower volume fractions, but at the time of this work, we do not have them available.

**Figure 21:**
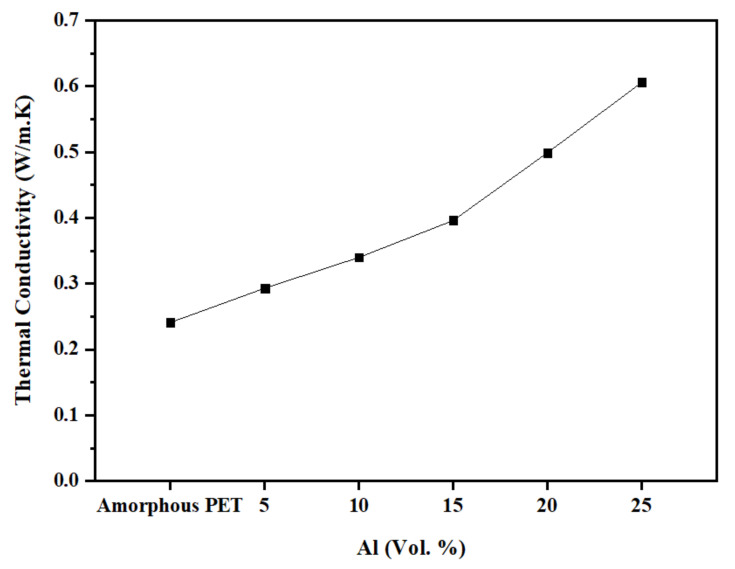
Thermal conductivity as a function of Al content.

The thermal conductivity values for the amorphous PET and the Al–PET composites are shown in Figure 21. A gradual increase in the thermal conductivity of the composites is observed with increase in the loading of the aluminium particles. Whereas with the electrical conductivity, we were just below the volume fraction for percolation, we were well below the threshold concentration where the thermal conductivity shoots up. Typically, the percolation threshold for thermal conductivity is higher than for electrical conductivity, and is over 60 vol.%. In the 25 vol.% Al composite, the thermal conductivity was over 2x the base material (0.606 W/m.K). Industrial heat-conducting plastics on the market have conductivities in the range of 0.4–40 W/m.K; hence, we are at the bottom end of the commercial conductive plastics. Again, with hot powder compaction, we could reach the loading levels (60 vol.%) needed for reaching the percolation threshold for thermal conductivity, but this is not possible with injection-moulding.

## 4. Conclusions

This work incorporated Al nano platelets in amorphous PET. There was a doubling in modulus, as seen with other rigid fillers, an increase in tensile strength, and a drop in elongation-to-break, which was even higher than with other filler-plastic composites, yet there was an increase in the notched Izod impact resistance. In the above regard, the trend observed with irregular nodular Al particles in amorphous PET extends to Al particles of other shapes. There have been other works using Al as a filler in plastics (with PP, PVC, PC and SAN), but the natural adhesion of Al–PET was not present, and hence, in those works, the conventional trend of increases in modulus, but with decreases in tensile strength, elongation-to-break, and impact resistance, were observed. The Al–PET combination does not follow this trend, allowing the simultaneous increase in tensile and flexural moduli, as well as in the strength and impact resistance.

The unusual combination of mechanical properties mentioned above is, thus, common to PET filled with Al particles of any shape. However, there are also differences caused by particle shape: Al platelets gave higher flexural moduli than nodular Al, and they influenced the appearance of the composite. The composites with nano Al platelets gave a flexural modulus reaching 8 GPa, which is about double of what is reported for other platelet materials such as talc, mica, and even graphene composites. The bars had a silvery appearance. Both these effects were due to the high orientation of the nano Al platelets by the flow. The Al platelets were ductile, and where they were too wide to fit in, they folded back on themselves, rather than break.

While we have concentrated on the mechanical robustness of Al-filled PET, its ultimate attraction is to secure a conductive plastic that is mechanically sound, easy to process, and not too expensive. In this work with Al nano platelets in PET, we fell a little short of the volume fraction for attaining electrical percolation, as we could not go above 25 vol.% due to the increase in melt viscosity. However, we have found it was possible to cross the percolation threshold with 25 vol.% Al platelets in a polyester blend (to be published). With PET, the way forward to reach the percolation threshold is by using either a mixture of Al platelets and spherical Al particles, or Al micro fibres. Future work will test this. Our work shows that the mechanical properties of PET will not be impaired by Al particles of any shape. Finally, to attain a commercially acceptable conductive plastic, flame retardant additives have to be incorporated, and this requires another round of iteration in the material development.

## Figures and Tables

**Table 1:** Physical properties of the aluminium flakes according to manufacturer (Nanografi Nano Technology, Germany).

Property	Al Powder
Particle size	Less than 45 μm (325 Mesh)
Purity	99.99%
Appearance	Grey powder
Morphology	Flakes
Density	2.7 g/cm^3^

**Table 2:** Al loading percentages and theoretical densities of Al/amorphous PET composites.

Sample ID	Al Loading (vol.%)	Corresponding Al Loading (Wt.%)	Theoretical Composite Density (g/cc)
0% Al 1/PET	0	0.00	1.333
5% Al 1/PET	5	9.63	1.401
10% Al 1/PET	10	18.37	1.469
15% Al 1/PET	15	26.33	1.538
20% Al 1/PET	20	33.62	1.606
25% Al 1/PET	25	40.30	1.674

**Table 3:** Thermal parameters obtained from the first cycle of DSC thermographs (Figure 6) of neat amorphous PET and Al/amorphous PET composites.

First Cycle							
Sample	T_g_	T_cc_	∆H_cc_	T_m_	∆H_m_	T_c_	∆H_c_
	°C	°C	J/g	°C	J/g	°C	J/g
Neat PET	75.1	141.1	32.7	246.2	−45.6	179.1	46.0
5% Al/PET	75.8	126.1	27.4	246.5	−43.6	201.8	40.6
10% Al/PET	76.0	124.4	22.9	245.2	−40.5	200.7	34.9
15% Al/PET	75.3	124.6	21.4	245.2	−37.1	201.8	32.6
20% Al/PET	75.8	117.4	19.4	243.8	−36.1	199.3	28.4
25% Al/PET	74.8	120.7	17.9	245.9	−33.6	195.6	24.5

**Table 4:** Mechanical properties of PET with various nano platelet fillers. The values in brackets are those of the control material (unfilled PET) as reported in the respective works. N.R.—Not Reported.

PET & Platelet Filler	% Platelet	Tensile Modulus (Gpa)	Tensile Strength (Mpa)	Elongation-to-Break (%)	Flexural Modulus (Gpa)	Flexural Strength (Mpa)	Flexural Strain at Break (%)	Notched Izod Impact (J/m)	Ref.
‘PET Alloy’ with Talc platelets	20 wt.% of talc	N.R.	N.R.	N.R.	4.93 (N.R.)	103.4 (N.R.)	3.8 (N.R.)	42.2 (N.R.)	[49]
Graphene–amorphous PET	10 wt.%	2.9 (1.9)	57 (54)	4(100)	N.R.	N.R.	N.R.	[Toughness 0.3 (720) kJ/m^3^]	[39]
Non-functionalised Graphene–PET	4 wt.%	1.82 (1.65)	N.R.	~80 (420)	N.R.	N.R.	N.R.	N.R.	[40]
Functionalised grapheneoxide in PET	0.5 wt.%	0.79 (0.49)	60 (30)	90 (60)	N.R.	N.R.	N.R.	N.R.	[48]
Untreated clay–PET	3%	N.R.	N.R.	N.R.	2.23 (1.7)	85 (76)	N.R.	N.R.	[50]
Mica nano platelet–PET composites	5%	2.68 (2.47)	40.3 (59.6)	2.4 (5.2)	N.R.	N.R.	N.R.	N.R.	[51]
Calcium terephthalate nano platelets + PET	20 wt.%	4.94 (2.89)	36.5 (41)	1.3 (244)	4.22 (2.91)	65 (113)	8 (15)	N.R.	[52]
Calcium terephthalate platelets + PET	30 wt.%	5.41 (2.89)	0.3 (41)	0.3 (244)	5.01 (2.91)	60 (113)	1.5 (15)	N.R.	[52]
Al nano platelet-PET	25 vol.%	3.1 (1.5)	70 (60)	1 (176)	8.03 (2.63)	103.4 (96.7)	2.09 (6.89)	29.4 (25.9)	This work

**Table 5:** For the unpaired t-test, the null hypothesis was that there is no difference between the means of the two compositions that are compared, and the alternative hypothesis applied was that the two means are different at the 5% significance level. ‘Significant’ means the alternative hypothesis is accepted and that there is a difference between the means, and ‘non-significant’ means we accept the null hypothesis that there is no difference in the means; ‘vs.’ stands for ‘versus’.

*t*-Test Comparison of Means of Izod Impact, for Two Compositions	*t*-Test Significance at 5% Level, for Difference of Means of Izod Impact Resistance
Any Al composition vs. Amorphous PET	Significantly Different
5 vol.% Al vs. 10 & 15 vol.% Al	Not significantly Different
5 vol.% Al vs. 20 & 25 vol.% Al	Significantly Different
10 vol.% vs. 15 vol.% Al	Not significantly Different
10 vol.% vs. 20 vol.% Al	Significantly Different
10 vol.% vs. 25 vol.% Al	Significantly Different
15 vol.% vs. 20 vol.% Al	Significantly Different
15 vol.% vs. 25 vol.% Al	Significantly Different
20 vol.% vs. 25 vol.% Al	Not significantly Different

## Data Availability

Not applicable.

## Supplementary Materials

The following supporting information can be downloaded at: https://www.mdpi.com/article/10.3390/polym14030630/s1, Figure S1: Wide angle X-ray diffractogram of the PET (pellets) which was extrusion compounded with the Al nanoplatelet powder, shows it was semi-crystalline, Figure S2: DSC thermogram of the PET pellet. The melting curve is typical of semi-crystalline PET, Figure S3: Wide angle X-ray diffractogram of the nanoplatelet Aluminium powder used in the work. Figure S4: Stress – Strain curves for amorphous PET (9 samples). Amorphous materials are prone to physical ageing, which causes a reduction in elongation-to-break with increasing storage time. Figure S5: Stress – Strain curves for 5 vol. % nanoplatelet Al-PET composite (5 samples). Figure S6: Stress – Strain curves for 10 vol. % nanoplatelet Al-PET composite (6 samples). Figure S7: Stress – Strain Curve for 15 vol. % nanoplatelet Al-PET composite (8 samples). Figure S8: Stress – Strain Curve for 20 vol. % nanoplatelet Al-PET composite (7 samples). Figure S9: Stress – Strain Curve for 25 vol. % nanoplatelet Al-PET composite (8 samples).
