# Supplementary material for: Amorphous Poly(ethylene terephthalate) Composites with High-Aspect Ratio Aluminium Nano Platelets
Source: Polymers (Basel). 2022 Feb 7;14(3):630. doi: 10.3390/polym14030630 (PMC8839713; doi:10.3390/polym14030630)
Supplement: Supplementary file 1 [file polymers-14-00630-s001.zip › polymers-1545902-SI.pdf]

Supplementary Information

Amorphous poly(ethylene terephthalate) composites with high aspect ratio Aluminium nano platelets

Experimental

Tensile testing

The tensile bars had the following dimensions: end-to-end length L = 150 mm, width W = 12.7 mm × Depth D (thickness) = 3.25 mm. The length of the narrow section of the bar was 57 mm and the gauge length was 55 mm. The radius of curvature of the curved portion (fillet) which joins the narrow part of the bar to the grip portion was 76 mm. Our tensile bar's dimensions correspond in all details to the Type I bar of ASTM D638-14 (Standard Test Method for Tensile Properties of Plastics), except in the ASTM Type I, the end-to-end length L = 165 mm. That is, in our bar, the grip section was shorter than in the ASTM bar by 7.5 mm, but this has no influence on the measurement, and as all other features including the gauge length are the same, we say our tensile bar was in essence the same as the Type I tensile bar of ASTM D638-14.

The cross-head speed used was 50 mm/min. ASTM D638-14 allows 5, 50 or 500 mm/min., and recommends the slowest according to the time to reach the extension to break (< 5 mins.). For amorphous PET if 5 mm/min. is chosen the extension-to-break would be 550 % and the time of the test would be too long. The intermediate value of 50 mm/min. was thus selected. This kept the extension-to-break for the amorphous PET under 10 minutes, and under 5 minutes for the Al-PETs (which had a greatly reduced extension-to-break). We are interested in the relative modulus (Al-PET's modulus/PET's modulus), hence a common cross head speed of 50 mm/min. was used for the PET and Al-PETs.

No humidity conditioning was used. The bars were prepared through injection moulding, stored in plastic bags and tested several days after moulding. They were kept in an air-conditioned laboratory at 20°C. PET is not as sensitive as polyamides and cellulose to moisture uptake. Again, this factor did not affect the relative modulus and the trend obtained was as expected. The number of samples was 9; all breaks were in the gauge length.

The tensile bar was injected from one end of the bar. The ASTM standard asks for any orientation effect to be noted. For the Al platelets, there would be anisotropy - in this case the platelets would align with the flow direction; that is edge-on if the tensile bar's section was viewed. This is discussed in the main text.

Flexural test

ASTM D790-03 ('Standard Test Methods for Flexural Properties of Unreinforced and Reinforced Plastics and Electrical Insulating Materials), Procedure A, was used to measure the flexural modulus and flexural strength of the unfilled and filled PETs.

Rectangular bars were injection moulded according to the dimensions of ASTM D790-03. As with the tensile tests, no humidity conditioning was used. The bar's length was 134 mm, the width was 12.7, and the depth (thickness) was 3.25 mm. The bars were tested flatwise rather than edgewise. The ASTM standard says that a support span-to-depth ratio of 16:1 shall be used. The span is the distance between the supports on which the bar rests. As the depth was 3.25 mm, the span was measured and set at 51.2 mm. As the bar's length was 134 mm, this left an overhang of 41.4 mm on either side of the support (ASTM D790-03 specifies an overhang of at least 10% of the support span which would be 5.12 mm, and no less than 6.4 mm). The span distance was kept constant for the PET and the PET bars with both types of Al powder. The bar was places on the supports such that the loading nose contacted in the middle of the bar. The radii of the loading nose and supports was 5.0 mm as specified in the standard. The cross-head speed was 2 mm/min, the calculated value of rate of crosshead motion was 1.365 mm/min. The ASTM D790-03 recommends a test speed range calculated depending on the support span, the depth of the beam and strain rate on the outer layer. The crosshead speed R should be set by $R = ZL^{2}/6d$; where L = support span in mm, d = depth of beam in mm, and Z is the rate of straining of the outer layer, in mm/mm/min. The standard specifies Z to be 0.01 and says that in no case shall the actual crosshead speed differ from that calculated from the equation, by more than 610 %. The calculated speed according to our choice of span (L = 51.2 mm) and depth (3.25 mm) was 1.365 mm/min., and our chosen speed of 2 mm/min. falls within the standard's range of 610% difference.

In composites, the orientation of the reinforcement can influence the flexural modulus and strength. The microscopy of the fractured tensile bars established they were seen only edge-on, implying they were nano platelets and were oriented with their flat surface parallel to the widest surface of the bar. The number of tests was 10 per composition. The tangent flexural modulus was calculated by drawing a tangent to the steepest initial straight-line portion of the load-deflection curve and using the following equation, $E_B = L^3 m/(4bd^3)$, where, E_B = modulus of elasticity in bending, MPa, L = support span, mm, b = width of bar tested, mm, d = depth of bar, mm, and m = slope of the tangent to the initial straight-line portion of the load-deflection curve, N/mm of deflection. No toe correction was applied.

The amorphous PET and PET bars with up to 15 vol. % for the nano platelets showed yielding behaviour, while higher volume fractions showed rupture characteristics, within the 5 % strain limit.

Notched Izod Impact Resistance

The impact resistance of the Al filled-PET bars was compared with the amorphous PET bar, by using the notched Izod test according to the ASTM D256-04. The moulded bar's dimensions were length of 64 mm, width of 12.7 mm and thickness of 3.25 mm as set by the standard. It was notched by machine. The V shaped notch was cut at the mid-point along the length of the bar (31.8 mm) and had full cone angle of 45 degrees and was 2.5 mm deep (radius). The distance in front of the notch in the bar was = 12.7-2.5 = 10.2 mm.

The standard has four methods of tests (A, C, D and E), and three types of break and one non-break as outcome. The standard says Test Method C is preferred over Test Method A for materials that have an Izod impact resistance of less than 27 J/m under the notch. In test method C a correction is applied to account for the toss energy (to propel the broken fragment) which is a substantial fraction of the energy for low impact samples. The amorphous PET had a notched Izod impact of ~ 25 J/m according to the literature, which is on the border between Type A and C; however, the Al-PET bars had values > 27 J/m, hence we decided to use Type A (no toss correction) for both amorphous PET and the Al-PET composites. The test was conducted notch wise (pendulum striking on the notch side).

The amorphous PET and all the Al-PET bars broke according to the type C (Complete Break) break mentioned in the standard - that is on break, the bar separated into two or more pieces. Hence, impact value comparisons are valid as the failure category was the same for all compositions.

The bars were stored for over 40 h at 23°C but not conditioned for humidity. At 23°C, PET is not as hygroscopic as polyamides and cellulose. Amorphous PET when stored for long periods does become more brittle due to physical ageing, but this would reduce the impact values. In the Al-PET, we generally saw an increase impact values, and this over rode the effect of physical ageing.

X-ray Diffractogram of PET Pellets

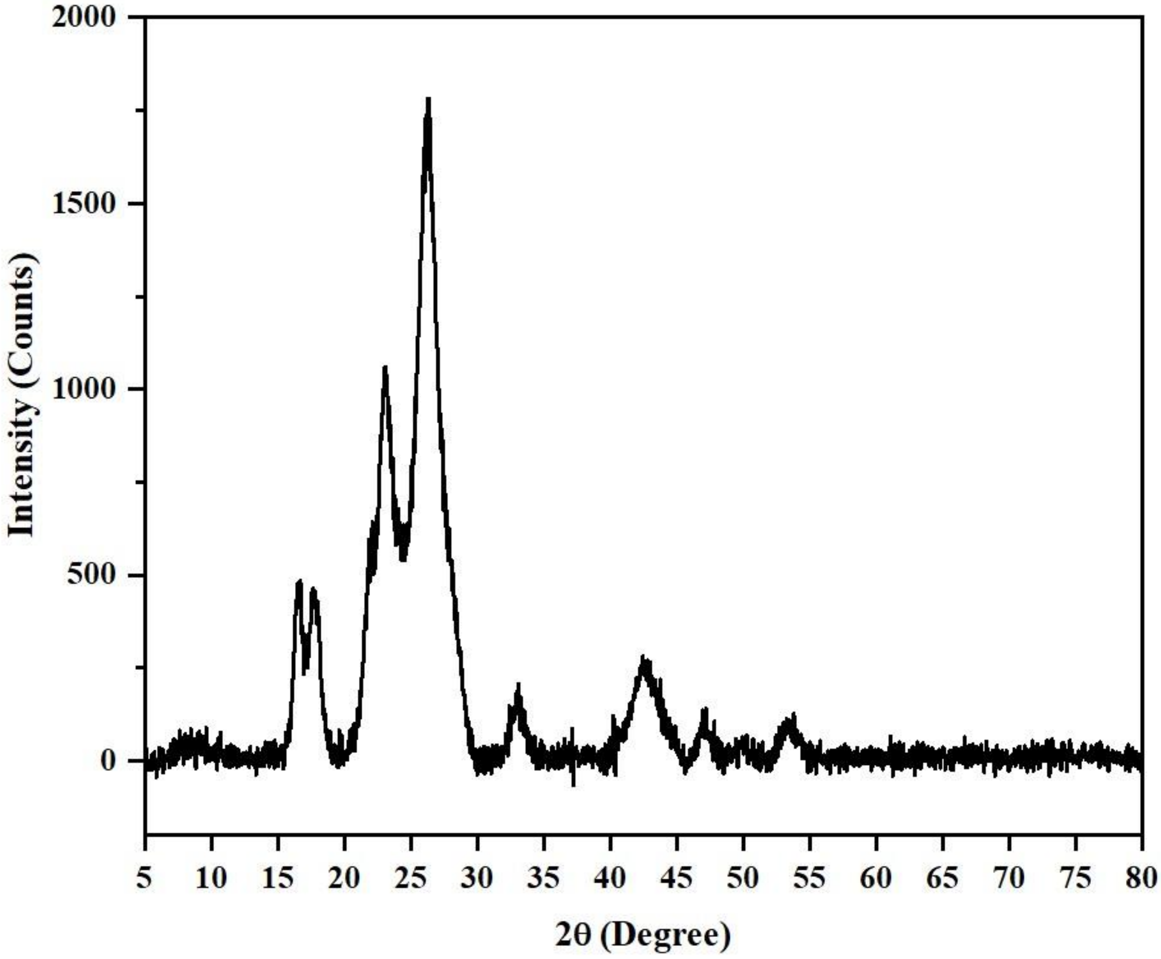

Figure S1. Wide angle X-ray diffractogram of the PET (pellets) which was extrusion compounded with the Al nanoplatelet powder, shows it was semi-crystalline. Compare with Figure 4 which shows the X-ray diffractograms of the amorphous PET and Al-PET bars after injection moulding with cold moulds

DSC of PET Pellets

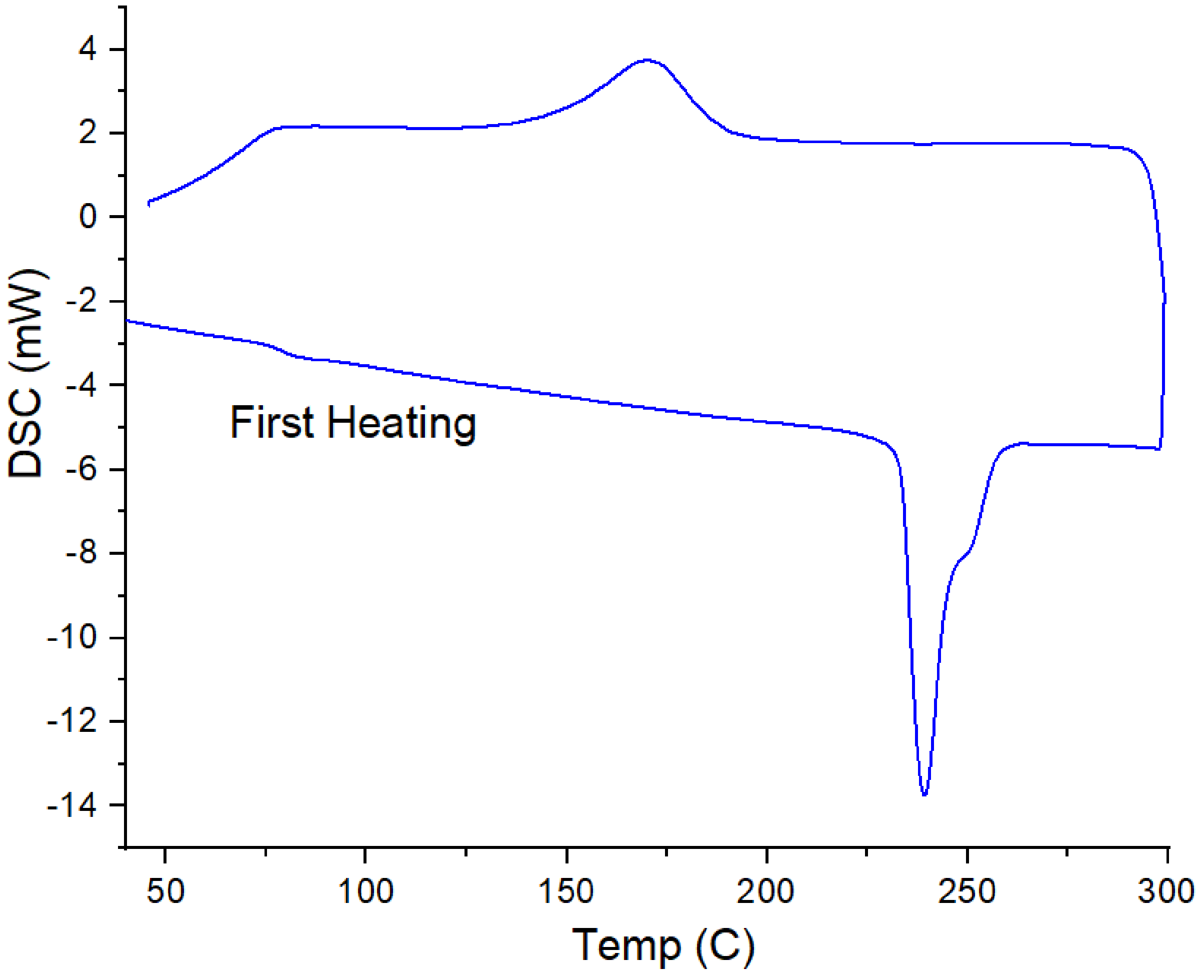

Figure S2. DSC thermogram of the PET pellet. The melting curve is typical of semi-crystalline PET. Compare with Figure 6 which shows the DSC of the amorphous PET and Al-PET bars

X-ay Diffractogram of Al nano Platelet Powders

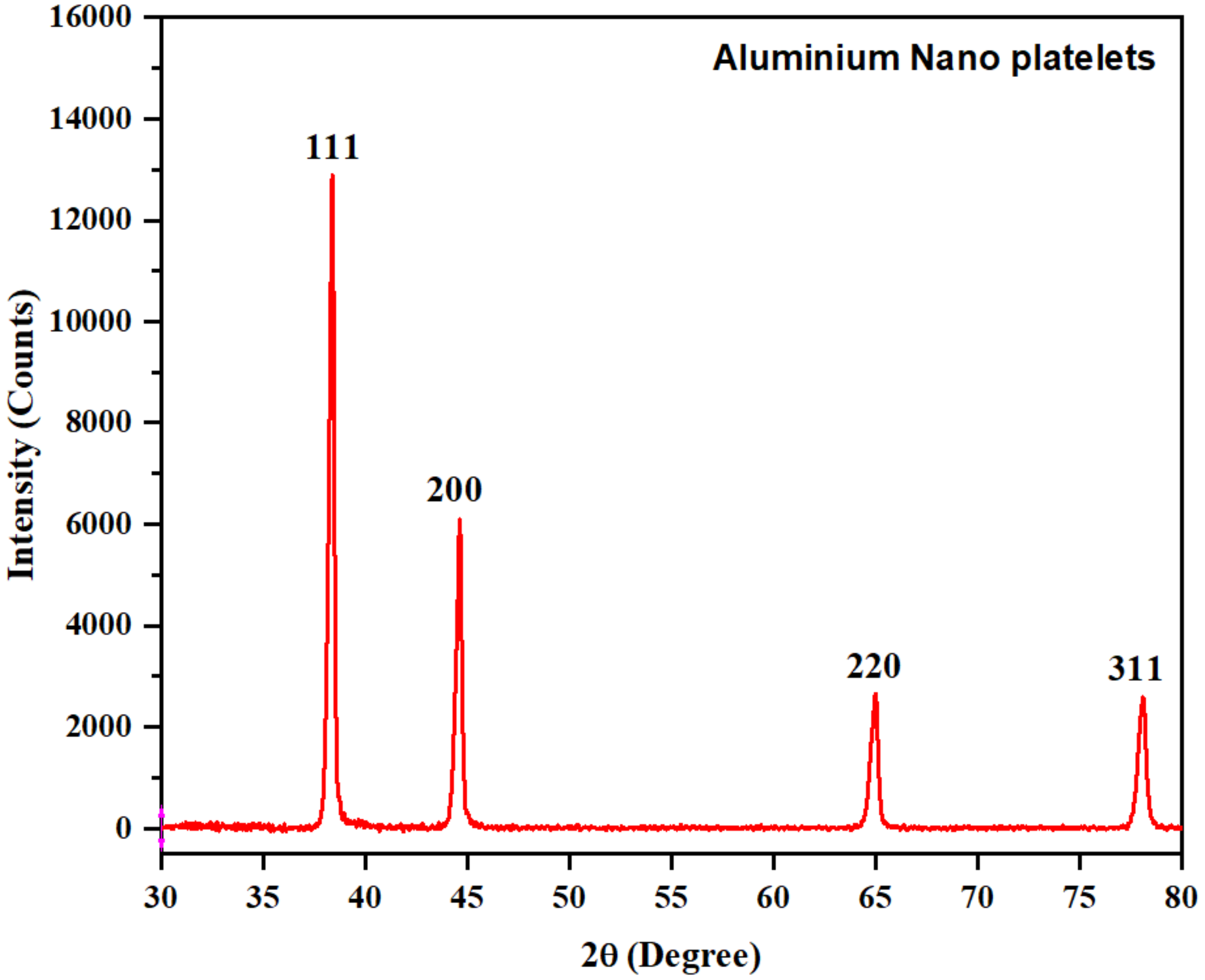

Figure S3. Wide angle X-ray diffractogram of the nanoplatelet Aluminium powder used in the work.

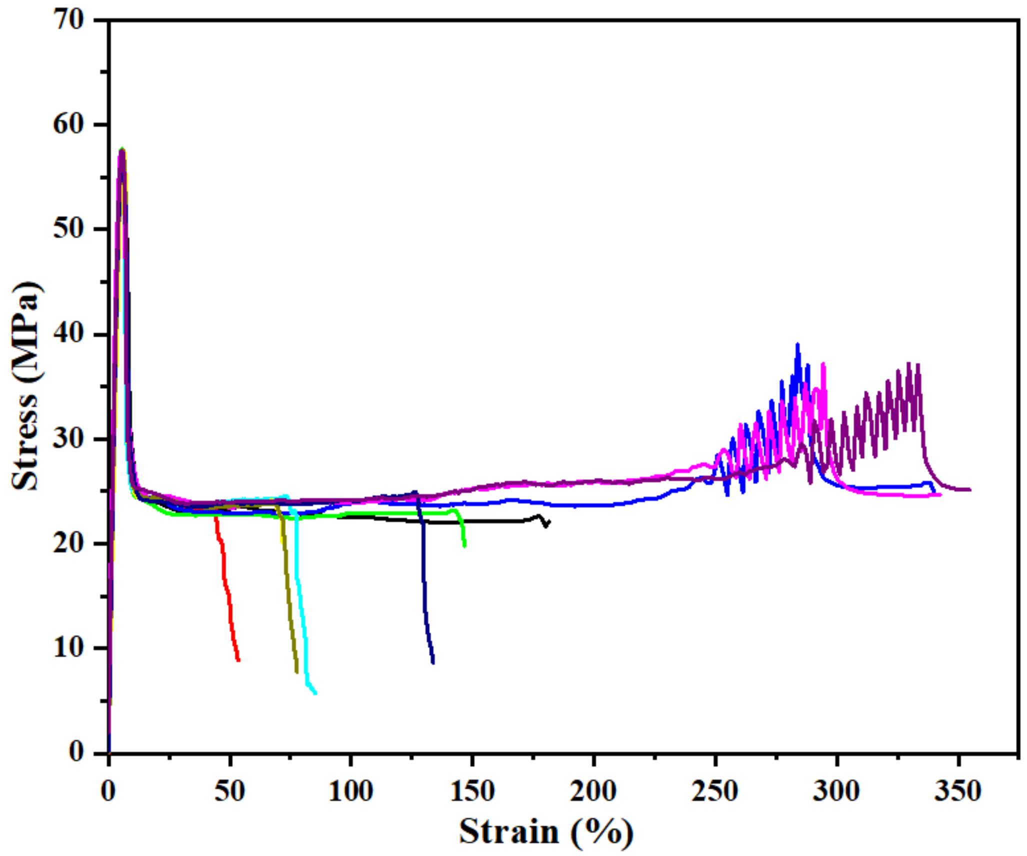

Figure S4. Stress – Strain curves for amorphous PET (9 samples). Amorphous materials are prone to physical ageing, which causes a reduction in elongation-to-break with increasing storage time. The relatively high variability in extension-to-break was due to physical ageing on storage; however, physical ageing does not cause high variability in the tensile modulus and strength. Amorphous PET shows necking with drop in the stress at about ~5% extension, and it can elongate to 550% if freshly made, but it would be lower after physical ageing. If extending beyond ~300%, strain hardening takes place with rise in stress, and this is due to the onset of oriented crystallisation; this is seen in the 3 curves that extend past 250%.

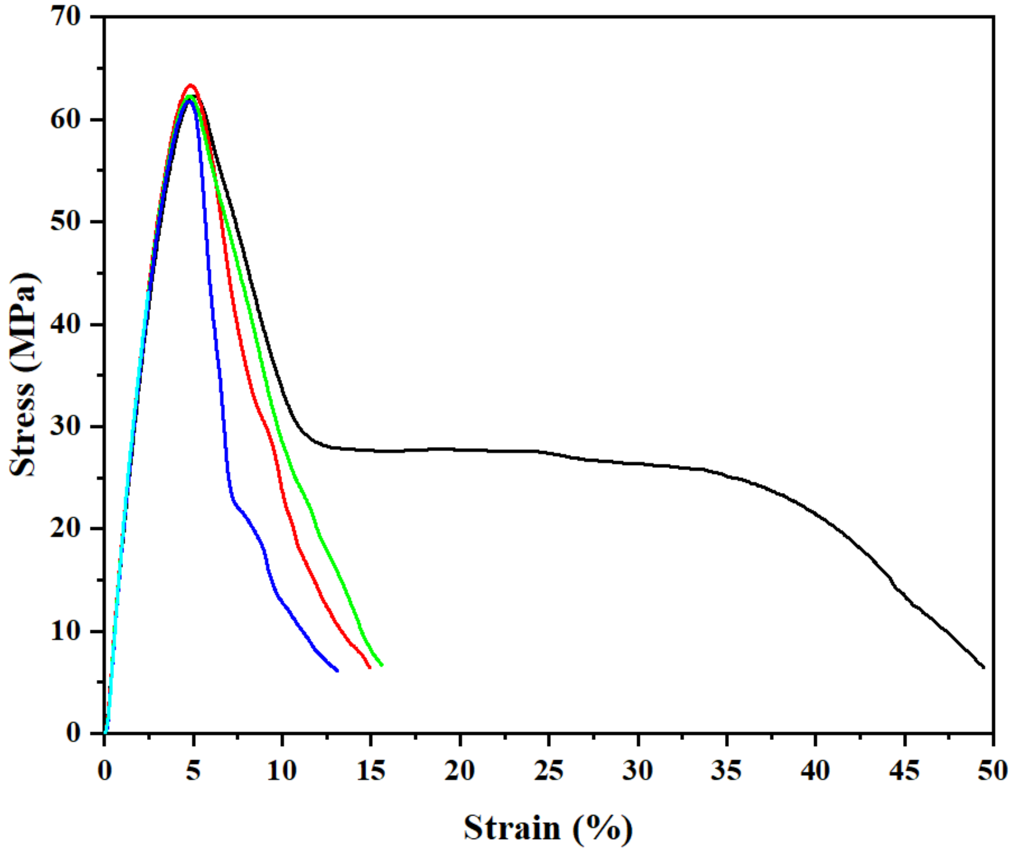

Figure S5. Stress – Strain curves for 5 vol. % nanoplatelet Al-PET composite (5 samples).

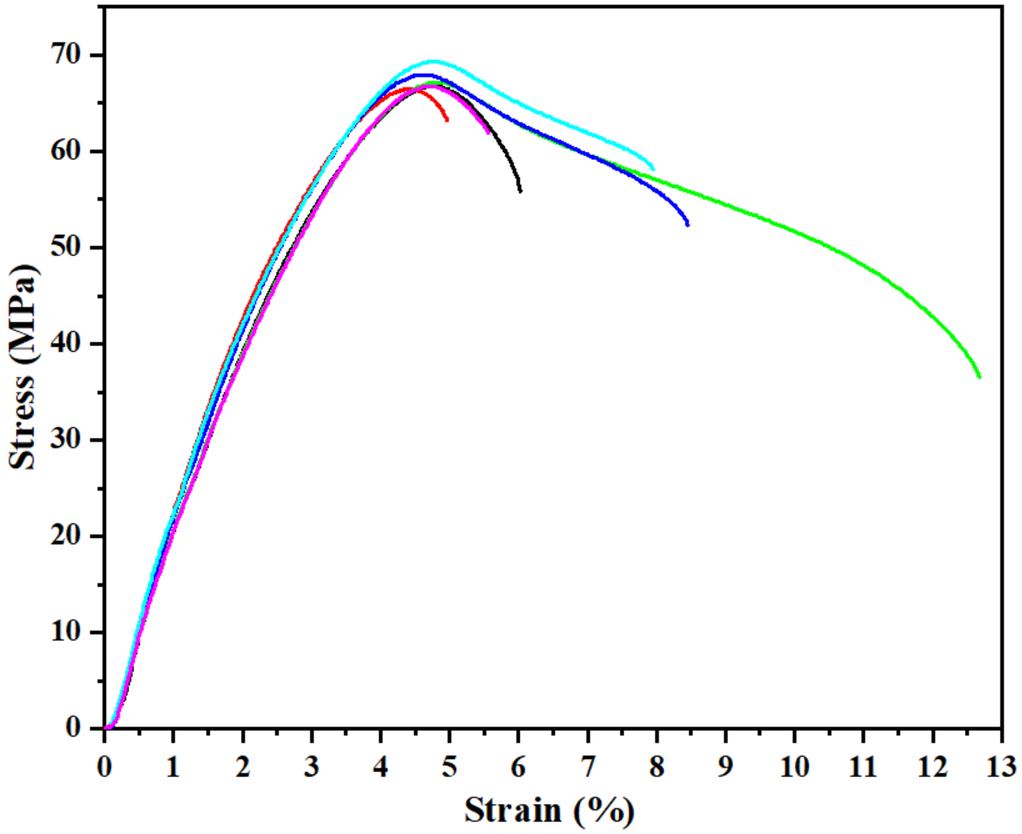

Figure S6. Stress – Strain curves for 10 vol. % nanoplatelet Al-PET composite (6 samples).

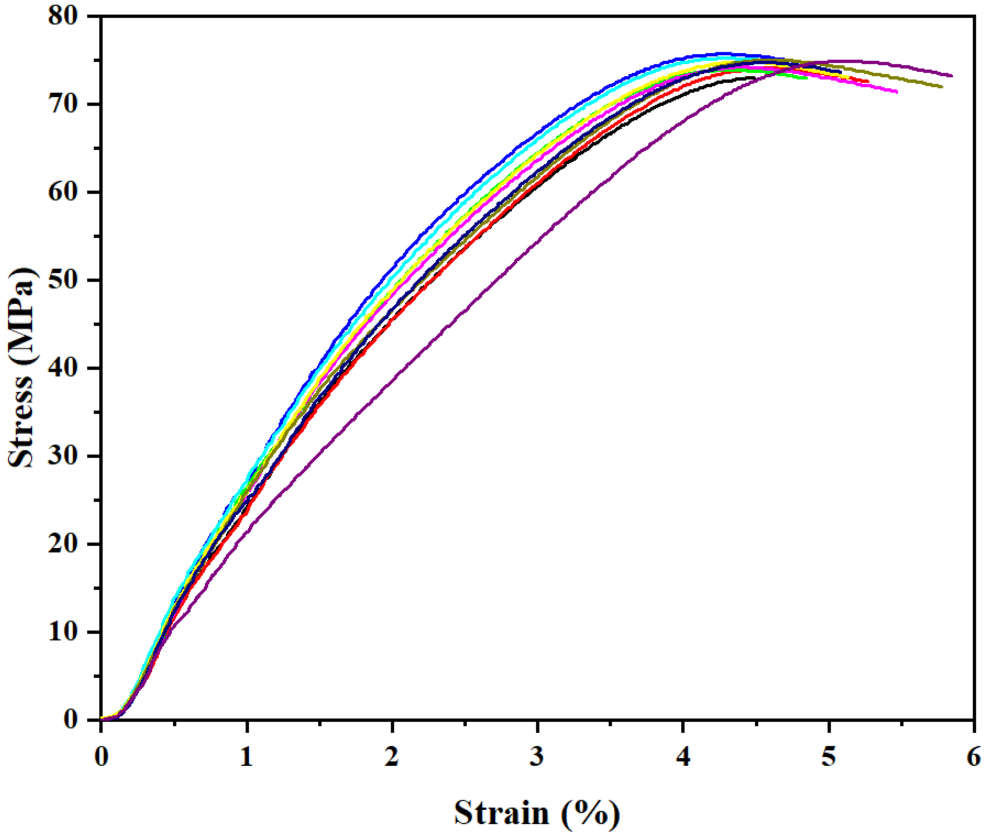

Figure S7. Stress – Strain Curve for 15 vol. % nanoplatelet Al-PET composite (8 samples).

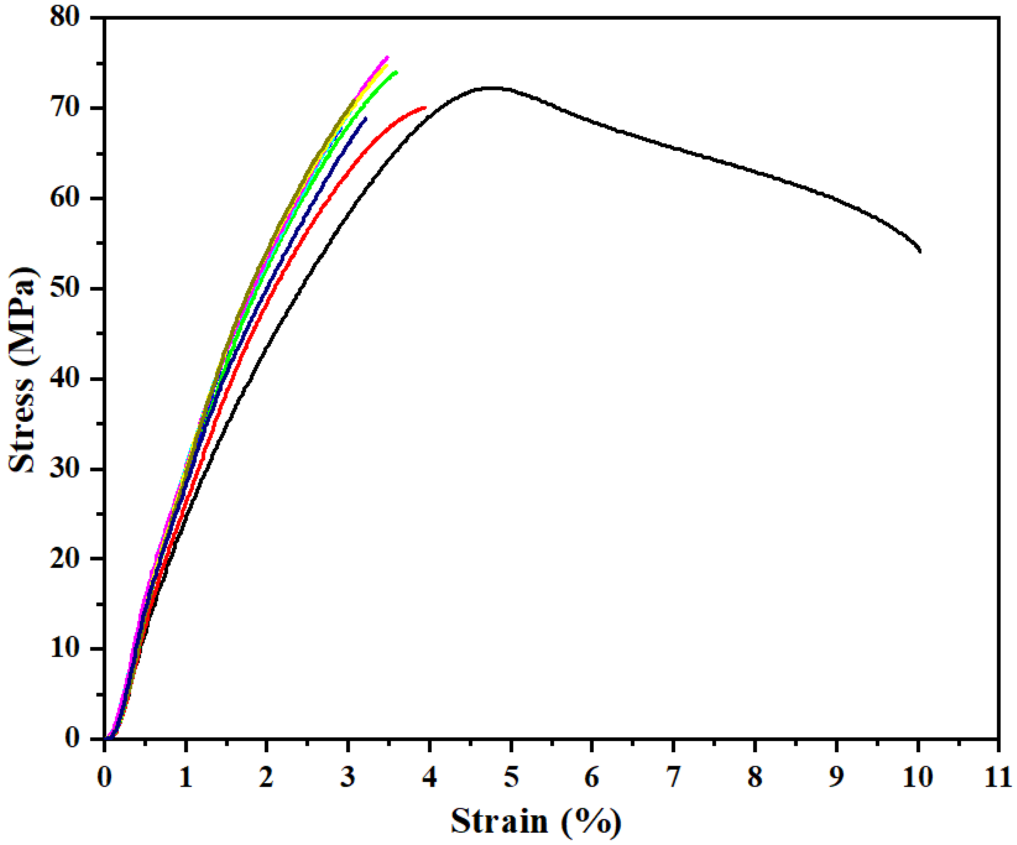

Figure S8. Stress – Strain Curve for 20 vol. % nanoplatelet Al-PET composite (7 samples).

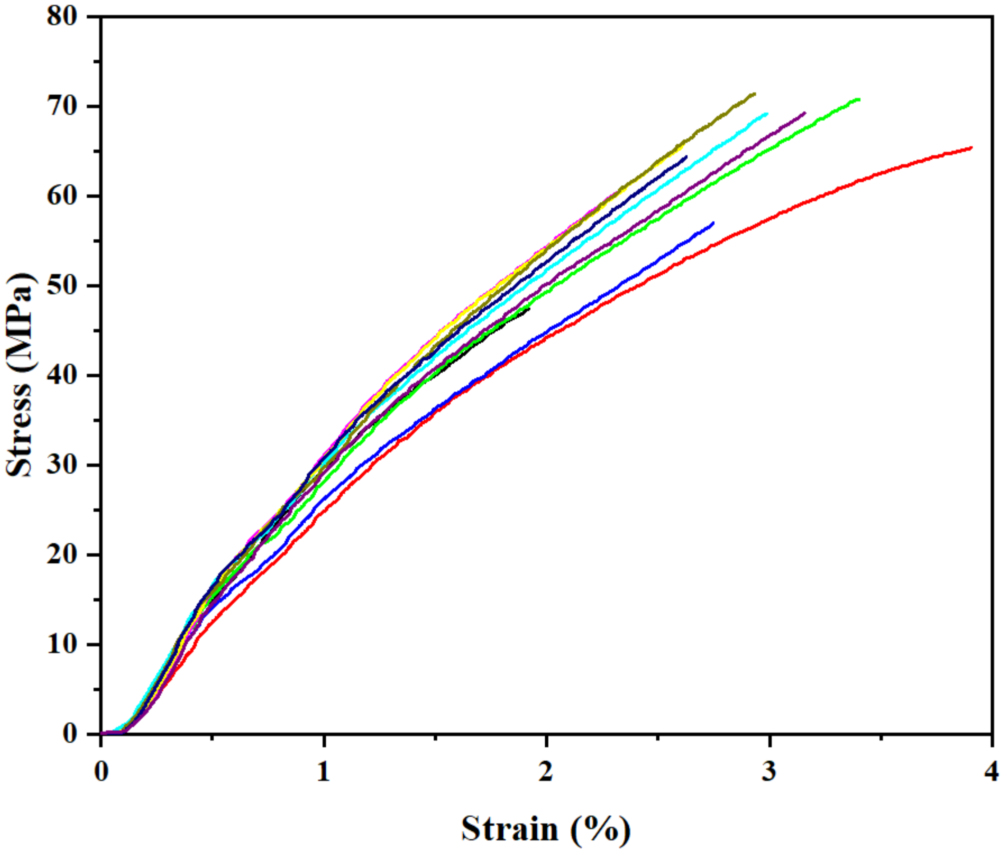

Figure S9. Stress – Strain Curve for 25 vol. % nanoplatelet Al-PET composite (8 samples).
